# Natural Variants of At*HKT1* Enhance Na^+^ Accumulation in Two Wild Populations of *Arabidopsis*

**DOI:** 10.1371/journal.pgen.0020210

**Published:** 2006-12-01

**Authors:** Ana Rus, Ivan Baxter, Balasubramaniam Muthukumar, Jeff Gustin, Brett Lahner, Elena Yakubova, David E Salt

**Affiliations:** Center for Plant Environmental Stress Physiology, Purdue University, West Lafayette, Indiana, United States of America; The Salk Institute for Biological Studies, United States of America

## Abstract

Plants are sessile and therefore have developed mechanisms to adapt to their environment, including the soil mineral nutrient composition. Ionomics is a developing functional genomic strategy designed to rapidly identify the genes and gene networks involved in regulating how plants acquire and accumulate these mineral nutrients from the soil. Here, we report on the coupling of high-throughput elemental profiling of shoot tissue from various *Arabidopsis* accessions with DNA microarray-based bulk segregant analysis and reverse genetics, for the rapid identification of genes from wild populations of *Arabidopsis* that are involved in regulating how plants acquire and accumulate Na^+^ from the soil. Elemental profiling of shoot tissue from 12 different *Arabidopsis* accessions revealed that two coastal populations of *Arabidopsis* collected from Tossa del Mar, Spain, and Tsu, Japan (Ts-1 and Tsu-1, respectively), accumulate higher shoot levels of Na^+^ than do Col-0 and other accessions. We identify At*HKT1,* known to encode a Na^+^ transporter, as being the causal locus driving elevated shoot Na^+^ in both Ts-1 and Tsu-1. Furthermore, we establish that a deletion in a tandem repeat sequence approximately 5 kb upstream of At*HKT1* is responsible for the reduced root expression of *AtHKT1* observed in these accessions. Reciprocal grafting experiments establish that this loss of At*HKT1* expression in roots is responsible for elevated shoot Na^+^. Interestingly, and in contrast to the *hkt1–1* null mutant, under NaCl stress conditions, this novel At*HKT1* allele not only does not confer NaCl sensitivity but also cosegregates with elevated NaCl tolerance. We also present all our elemental profiling data in a new open access ionomics database, the Purdue Ionomics Information Management System (PiiMS; http://www.purdue.edu/dp/ionomics). Using DNA microarray-based genotyping has allowed us to rapidly identify At*HKT1* as the casual locus driving the natural variation in shoot Na^+^ accumulation we observed in Ts-1 and Tsu-1. Such an approach overcomes the limitations imposed by a lack of established genetic markers in most *Arabidopsis* accessions and opens up a vast and tractable source of natural variation for the identification of gene function not only in ionomics but also in many other biological processes.

## Synopsis

Unlike most animals, plants are sessile and cannot leave a poor-quality environment after germinating. They therefore need to tolerate the particular conditions they encounter to survive. This makes plants an ideal system for the study of adaptive variation, and this is particularly true of *Arabidopsis thaliana (Arabidopsis),* which shows substantial natural variation and for which numerous genetic tools exist. Using a combination of analytical chemistry, genetics, and genomics, the authors were able to identify the specific genetic alteration that drive the natural variation in shoot sodium (Na^+^) accumulation capacity observed in *Arabidopsis* populations from coastal regions of Spain and Japan (Tossa del Mar and Tsu, respectively). They observed that a deletion in the DNA responsible for regulating the expression of *HKT1*, a gene known to encode for a Na^+^ transporter, causes reduced expression of At*HKT1* in roots of both the Spanish and Japanese populations. Such altered expression results in the elevated shoot Na^+^ observed in these two populations. Interestingly, this novel version of the *HKT1* genes is also associated genetically with the enhanced NaCl resistance they observe in the Japanese population.

## Introduction

Plants are sessile and therefore have developed mechanisms to adapt to their environment, including the soil mineral nutrient composition. High-throughput elemental profiling of *Arabidopsis thaliana (Arabidopsis)* has been used in an effort to identify the genes and gene networks involved in regulating how plants acquire and accumulate mineral nutrients and trace elements from the soil [1]. In 2003, Lahner et al*.* [1], in a screening of 6,000 fast-neutron–mutagenized *Arabidopsis* plants grown under unstressed conditions, identified 51 mutants with altered shoot elemental profiles, and they estimated that about 2% to 4% of the *Arabidopsis* genome is involved in regulating the elemental composition or “ionome” of *Arabidopsis* (for review, see [2]), including accumulation of macronutrients, micronutrients, and nonessential elements such as Na^+^. Recently, one of these ionomic mutants was shown to harbor a deletion in At*HKT1* that is responsible for the elevated shoot Na^+^ phenotype of this mutant [3].

As an alternative to induced mutations (fast-neutron, ethylmethane sulfonate, etc.), the large reservoir of natural variation that exists in *Arabidopsis* is also a potentially powerful resource for the investigation of ionomic gene function [4–6]. Such natural variation has the advantage over induced mutations in that uncovering the adaptive significance of such variation provides tools for the integration of gene function in the context of whole plant physiology. However, this genetic resource is still underexploited, mainly because natural phenotypic variation is usually the result of genotypic variation at multiple loci. Even when dealing with monogenic traits, it is a major challenge to identify a particular gene controlling a phenotype of interest. Currently, fewer than ten genes have been identified in *Arabidopsis* using the natural variation approach [7,8], whereas variation in multiple traits such as floral and meristem development, resistance, and defense against pathogens as well as metabolic enzymes ([9] and references therein) have been documented. Genetic differences between local populations are presumably associated with adaptation to the prevailing environmental conditions, although well-established examples of this in *Arabidopsis* are limited. Such investigations are impeded by the fact that very little information exists about the environmental conditions and habitat of the *Arabidopsis* accessions that have been collected and are curated at the *Arabidopsis* Biological Resource Center. The term “accession” is used to refer to natural genetic variants of *Arabidopsis* to avoid the term “ecotype,” which would allude to a variant genetically adapted to a particular habitat [10,11].

Here, we have coupled high-throughput elemental profiling of shoot tissue [1] from various *Arabidopsis* accessions with DNA microarray-based bulk segregant analysis (BSA) [12,13] and reverse genetics, for the rapid identification of genes involved in regulating how plants acquire and accumulate Na^+^ from the soil. Such an approach using DNA microarray-based genotyping overcomes the time constraints imposed on using natural variation by the rapid identification of genetic markers, and opens up a vast and tractable source of natural variation for the identification of gene function not only in ionomics but also in many other biological processes. In a system with multiple quantitative trait loci, an alternative method, eXtreme Array Mapping, was developed to identify major quantitative trait loci using pools of lines with extreme phenotypes [14].

Using this approach, we have identified a novel At*HKT1* allele from two different *Arabidopsis* accessions collected from Tossa del Mar (Ts-1) and Tsu (Tsu-1), sites located on the coastal regions of Spain and Japan, respectively. This novel At*HKT1* allele, in both Ts-1 and Tsu-1, is responsible for the higher shoot Na^+^ accumulation observed in these accessions compared with the reference accession Col-0, when grown under background NaCl levels. Previously, expression of At*HKT1* has been shown to be localized in the vascular tissues throughout the plant, with expression being highest in the roots [15–17]. The *hkt1* null mutation results in an alteration of the Na^+^ distribution in the plant, with higher levels of Na^+^ accumulation in shoots and lower accumulation in roots compared to wild-type plants. Loss of At*HKT1* expression in the null *hkt1* mutant also confers sensitivity to high levels of NaCl [17–20]. All these studies are consistent with the current model that HKT1 functions to tightly regulate Na^+^ accumulation in shoots by unloading Na^+^ from the xylem in both roots and shoots and recycling Na^+^ to the roots via the phloem.

Results reported here confirm that this new naturally evolved At*HKT1* allele is responsible for the elevated shoot Na^+^ accumulation we observe in Ts-1 and Tsu-1, via the specific downregulation of At*HKT1* expression in roots. Reciprocal grafting also confirms that the primary site for HKT1 function in regulating shoot Na^+^ is in the root, with HKT1 playing little role in the shoot. Further, we suggest a deletion in the promoter region as the factor responsible for driving the differential expression of At*HKT1* in both Ts-1 and Tsu-1. Interestingly, and contrarily to the loss-of-function mutants *hkt1,* this novel allele appears to also be associated with enhanced NaCl tolerance.

## Results

### Ts-1 and Tsu-1 Accumulate Higher Levels of Na^+^ in the Shoot than Col-0

**Figure 1:**
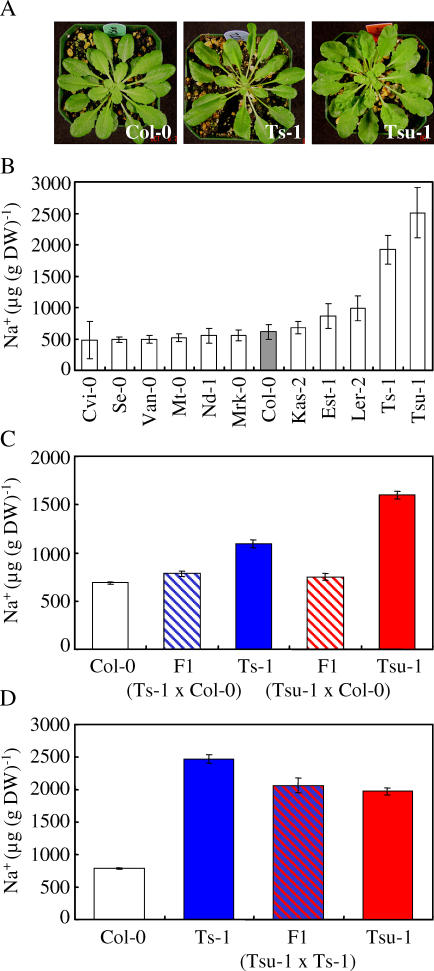
Genetic Analysis of the High Na^+^ Trait in Shoots of Arabidopsis thaliana Accessions Ts-1 and Tsu-1 (A) Seven-week-old plants of Col-0, Ts-1, and Tsu-1 accessions grown under short-day conditions. (B) Na^+^ levels in the shoot are higher in Ts-1 and Tsu-1. Na^+^ content in shoots of Cvi-0 (*n* = 12), Se-0 (*n* = 12), Van-0 (*n* = 12), Mt-0 (*n* = 12), Nd-1 (*n* = 12), Mrk-0 (*n* = 12), Col-0 (*n* = 12), Kas-2 (*n* = 12), Est-1 (*n* = 12), Ler-2 (*n* = 12), Ts-1 (*n* = 12), and Tsu-1 (*n* = 8). Presented data are the mean ± SD. (C) The higher Na^+^ accumulation in the shoot of Ts-1 and Tsu-1 is a recessive trait. Na^+^ content in shoots of Col-0 (*n* = 40), Ts-1 (*n* = 20), Tsu-1 (*n* = 20), and F1 plants derived from the crosses Ts-1 × Col-0 (*n* = 19) and Tsu-1 × Col-0 (*n* = 13). Presented data are the mean ± SE. (D) Ts-1 and Tsu-1 are allelic for the loci responsible for the higher Na^+^ levels in the shoot. Na^+^ content in shoots of Col-0 (*n* = 140), Ts-1 (*n* = 40), Tsu-1 (*n* = 40), and F1 hybrid plants derived from the cross Tsu-1 × Ts-1 (*n* = 10). Presented data are the mean ± SE.

Elemental profiling of shoot tissue collected from 12 different *Arabidopsis* accessions, including Col-0, Cvi-0, Est-1, Kas-2, Mrk-0, Mt-0, Se-0, Ts-1, Van-0, Nd-1, Tsu-1, and L*er*-2, revealed that Ts-1 and Tsu-1 (Figure 1A) accumulate higher levels of Na^+^ than Col-0 and all other accessions tested when propagated in soil with low levels of NaCl under controlled growth conditions (Figure 1B). Interestingly, the Ts-1 and Tsu-1 accessions were collected from the coastal regions of Spain (Tossa del Mar) and Japan (Tsu), respectively. Besides being annotated as having different geographical origins, Ts-1 and Tsu-1 have different physical appearances (Figure 1A). Moreover, based on a genome-scale DNA polymorphism analysis involving 96 *Arabidopsis* accessions, these two accessions appeared to be genetically distinct [21]. The Ts-1 and Tsu-1 accessions can also be distinguished based on the fact that Ts-1 accumulates elevated concentrations of cobalt (+55%) in shoot tissue compared to Col-0, while Tsu-1 has lower levels of Co. A complete set of the elemental profiling data summarized here can be obtained at the open access Purdue Ionomics Information Management System (PiiMS) at http://www.purdue.edu/dp/ionomics.

### Genetic Analysis and Mapping of the High Na^+^ Trait

**Figure 2:**
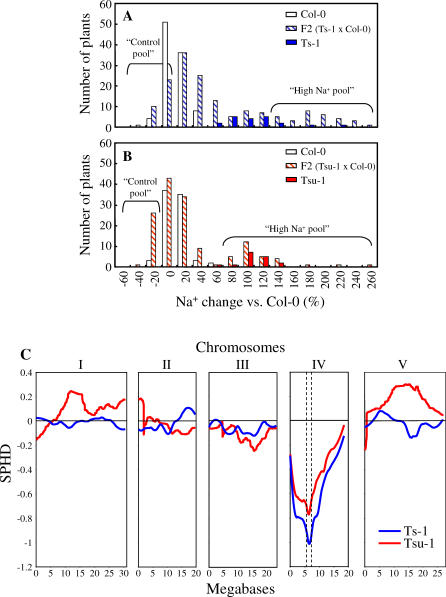
The Higher Na^+^ Accumulation in Shoots of Ts-1 and Tsu-1 Is a Monogenic Recessive Trait Caused by a Gene Located on Chromosome IV (A) Distribution of Na^+^ accumulation in shoot tissue of F2 segregating population obtained from crossing Col-0 with Ts-1. Presented data are distribution of Col-0 (*n* = 99), Ts-1 (*n* = 20), and F2 (Ts-1 × Col-0) (*n* = 158) plants. (B) Distribution of Na^+^ accumulation in shoot tissue of F2 segregating population obtained from crossing Col-0 with Tsu-1. Presented data are distribution of Col-0 (*n* = 80), Tsu-1 (*n* = 16), and F2 (Tsu-1 × Col-0) (*n* = 143). Na^+^ contents were calculated for each plant as a percentage relative to Col-0 average Na^+^ content. The “Control pool” and “High Na^+^ pool” labels indicate the F2 plants used to prepare the corresponding DNA pools for the DNA microarray-based BSA. (C) Hybridization of genomic DNA from Ts-1 (blue) and Tsu-1 (red) to DNA microarray (ATH1). Data are presented as a Scaled pool hybridization difference (SPHD), the difference between the hybridization of the two pools at the SFPs, scaled so that the difference between Col-0 and the accession would be equal to 1. SFPs were selected based on their *D*-statistic, which is a modified *t*-statistic that avoids spurious large values due to low hybridization levels [12]. Dashed vertical lines on Chromosome IV represent the mapping confidence interval of 0.875 Mbp on either side of the peak. Confidence intervals were calculated using algorithms derived from simulations by Borevitz et al. [12], and the scripts accessed at http://www.naturalvariation.org.

To determine the feasibility of using natural variation to identify individual loci regulating ionomic variation, we attempted to map the locus responsible for elevated Na^+^ in Ts-1 and Tsu-1. Both accessions were crossed to Col-0, and the shoot concentrations of Na^+^ were measured in the F1 hybrid plants (Figure 1C). The concentration of Na^+^ was not significantly different from Col-0, suggesting that the loci controlling Na^+^ accumulation in Ts-1 and Tsu-1 are recessive in both cases. Analysis of the segregating F2 plants from either cross revealed a pattern consistent with 3:1 segregation ratio of Col-0 × Ts-1 (Figure 2A) and Col-0 × Tsu-1 (Figure 2B) for shoot Na^+^ concentrations. Therefore, we conclude that the high Na^+^ phenotype observed in both Ts-1 and Tsu-1 segregates as a monogenic recessive trait. We further used the complementation test to demonstrate that the loci driving elevated shoot Na^+^ in Ts-1 and Tsu-1 are allelic to each other, showing that the F1 plants from Tsu-1 × Ts-1 have shoot Na^+^ levels that are similar to each parent (Figure 1D). Based on this evidence, DNA microarray-based BSA [12,13] was used to map the locus responsible for elevated Na^+^ in both Ts-1 and Tsu-1. From the Ts-1 × Col-0 F2 population represented in Figure 2A, two pools of DNA were prepared, one containing genomic DNA from 30 plants with Na^+^ levels similar to Col-0, constituting the “control” pool, and another with DNA from 30 plants with the highest Na^+^ levels, similar to those of the Ts-1 parent, constituting the “high Na^+^” pool. Single feature polymorphisms (SFPs) were identified for Col-0 and Ts-1 by comparing the hybridization of genomic DNA isolated from the parental Col-0 and Ts-1 accessions to the Affymetrix *Arabidopsis* ATH1 microarrays. The frequency of these SFPs in the DNA from the pool of F2 progeny plants with high Na^+^ levels, and in the pool with control Na^+^ levels, was scored by hybridization of genomic DNA to ATH1 microarrays. The DNA microarray-based BSA revealed a strong peak and therefore enrichment in Ts-1 SFPs linked to the locus causing elevated shoot Na^+^ in Ts-1 at 6.6 Mbp on Chromosome IV (Figure 2C). Similarly, a strong peak at 6.4 Mbp in Chromosome IV was obtained when the hybridization was performed using DNA pools from the Tsu-1 × Col-0 F2 population (Figure 2C). Approximately 513 genes (corresponding to 23 BAC clones) are contained within the confidence interval of 5.525 to 7.475 Mbp on Chromosome IV. The confidence interval was derived using previously published simulations [12]. One of the genes in BAC clone F24G24 (at 6.39 Mb), At4g10310, named At*HKT1,* stands out as a very strong candidate because it has been previously identified as a Na^+^ transporter [15], which when disrupted causes elevated shoot Na^+^ [3,17,18]. The shoot Na^+^ concentrations in the *hkt1–1* null mutant are significantly higher than Col-0 in leaves at all stages of development, with Na^+^ accumulation generally increasing with leaf age (Figure S1A). When grown under the same conditions, the Ts-1 and Tsu-1 accessions also show increased Na^+^ accumulation compared to Col-0 in leaves at all stages of development. However, Na^+^ accumulation in Ts-1 and Tsu-1 is lower than that observed for *hkt1–1* (Figure S1A).

### The Ts-1 and Tsu-1 Alleles of At*HKT1* Have Similar Polymorphisms Compared to the Col-0 Allele

To establish if a different allele of At*HKT1* is present in Ts-1 and Tsu-1, the nucleotide sequence was determined for the At*HKT1* locus in both accessions. The sequenced region comprises 5,456 bp upstream (363 bp downstream of the previous gene At4g10300) of At*HKT1* translational start codon that includes the promoter to 386 bp downstream of the At*HKT1* stop codon. Several polymorphisms were identified both in the coding region and in the upstream and downstream regions of the At*HKT1* gene. In the coding region, no nonsense mutation was recognized and 19 single nucleotide polymorphisms (SNPs) were identified in both Ts-1 and Tsu-1 (Figure S2A), seven of which result in the amino acid changes R3I, K10N, V66L, C134Y, E385G, V453L, and F477L. None of the amino acid changes are located in the three relatively highly conserved regions identified by Uozumi et al. [15], and none of them affect the first serine residue at position 68 of P-loop A that has been implicated in the Na^+^ specificity of the transporter [22]. One of the amino acid changes, valine to leucine in position 453 (V453L), was also found in the L*er* At*HKT1* allele published by Uozumi et al. [15].

In the upstream region, which includes the promoter of At*HKT1*, four major polymorphisms were identified between Ts-1, Tsu-1, and Col-0 (Figure S2B). The most upstream polymorphism affects two repeated units of 681 and 673 bp, linked by a 34-bp sequence (Figure S2B). These two repeated units are localized 5,320 to 3,933 bp upstream of the At*HKT1* start codon and are 97% identical to each other in Col-0. In Ts-1 and Tsu-1, we identified a 725- and 687-bp deletion, respectively, which removes the end of the first of these repeated units, the beginning of the second unit, and the 34-bp junction sequence. Consequently, only one of the repeated units is present in Ts-1 and Tsu-1. The second major polymorphism identified in this region, about 3,100 bp upstream of the start codon, is an insertion of 13 bp in the sequence of Ts-1 as well as Tsu-1, resulting in the duplication of a 10-bp sequence (AATGTGTTAT) in Ts-1 and Tsu-1 that is present only once in Col-0. Similarly, 1,210 bp upstream of the start codon, a 14-bp insertion results in a duplicated 10-bp sequence (TCATTGCAAA) in Ts-1 and Tsu-1. The last major polymorphism in the promoter region is localized 148 to 98 bp upstream of the translational start codon. Compared to Col-0, these sequence polymorphisms found in both Ts-1 and Tsu-1 abolish the first of the two putative CAAT boxes suggested by Uozumi et al. [15].

Polymorphisms have also been found in the two introns of At*HKT1*, mainly SNPs. We have been unable to sequence the last 157 bp of the second intron of the Ts-1 and Tsu-1 alleles, which according to the published Col-0 sequence is enriched in [TA] repeats. Finally, major differences in the sequence between Col-0 and Ts-1 and Tsu-1 occur in the sequence downstream of the stop codon, including the 3′-untranslated region. Several SNPs, as well as deletions in Ts-1 and Tsu-1, result in a sequence of 246 and 140 bp shorter than the 386-bp sequence found in Col-0.

### Complementation Studies Indicate At*HKT1* Is Responsible for the High Na^+^ Content in Shoots of Ts-1 and Tsu-1

**Figure 3:**
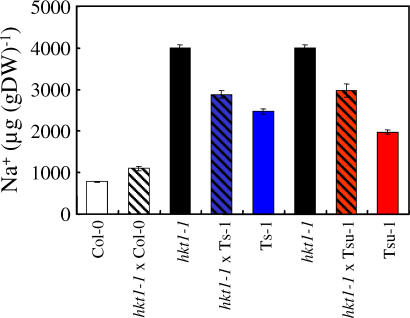
Complementation Studies Indicate At*HKT1* Natural Variant Is Responsible for the Higher Na^+^ Content in Shoots of Ts-1 and Tsu-1 Na^+^ contents were analyzed in shoots of Col-0 (*n* = 140), *hkt1–1* (*n* = 78), Ts-1 (*n* = 40), Tsu-1 (*n* = 40), and F1 hybrid plants derived from the crosses of *hkt1–1* × Col-0 (*n* = 12), *hkt1–1* × Ts-1 (*n* = 50), and *hkt1–1* × Tsu-1 (*n* = 10). Plants were grown for 42 d under short-day conditions. Presented data are the mean ± SE.

Because the Ts-1 and Tsu-1 *HKT1* alleles are recessive (Figure 1C) and the *hkt1–1* null mutant, like Ts-1 and Tsu-1, shows elevated shoot Na^+^ accumulation (Figure S1), we used the deficiency complementation test to establish that At*HKT1* is the gene responsible for elevated Na^+^ in Ts-1 and Tsu-1 [23]. Both accessions, Ts-1 and Tsu-1, were crossed to the *hkt1–1* T-DNA insertional mutant in the Col-0 *gl1* genetic background [19]. The *hkt1* null mutant accumulates higher levels of Na^+^ in the shoot and lower levels in the root than the wild-type, implicating HKT1 in the regulation of Na^+^ distribution throughout the plant [17–19]. F1 plants from the cross of *hkt1–1* to Col-0 present Na^+^ levels similar to the those of the wild-type, as expected from a recessive mutation (Figure 3). However, F1 hybrid plants from the crosses of *hkt1–1* (Col-0 *gl1*) with Ts-1 or Tsu-1 accumulate levels of Na^+^ that are intermediate between each parent and significantly higher than Col-0 (Figure 3), indicating that the Ts-1 and Tsu-1 *HKT1* allele is deficient in complementing the *hkt1–1* null mutation. This helps establish that elevated Na^+^ in Ts-1 and Tsu-1 is due to alterations in the At*HKT1* gene. However, the lower levels of Na^+^ in the F1 plants compared with *hkt1–1* suggest that the Ts-1 and Tsu-1 alleles of At*HKT1* are not nulls but rather weak alleles, although a role of the genetic background cannot be excluded (Figure 3).

### The Weaker Ts-1 and Tsu-1 At*HKT1* Allele Is the Result of Its Reduced Expression in the Root

**Figure 4:**
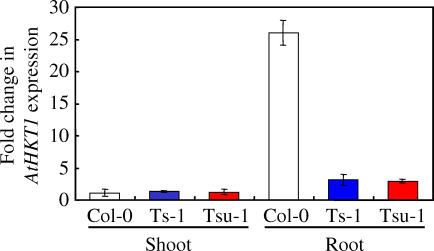
Root Differential At*HKT1* Expression between Col-0, Ts-1, and Tsu-1 At*HKT1* level of expression was compared in shoots and roots of Col-0, Ts-1, and Tsu-1 plants. RNA was isolated from shoot and root of 60-d-old plants grown in soil under short-day conditions. For normalization across samples, the expression of the *Actin 1* gene was used, and relative fold induction was calculated in comparison to At*HKT1* expression in Col-0 shoot using the ΔΔ*C*_t_ method. Presented data are the means of at least three biological replicates, and the error bars represent ±SD.

Several polymorphisms in the promoter region suggested that *HKT1* in Ts-1 and Tsu-1 might have a different expression level or expression pattern compared to Col-0, which could be responsible for the high Na^+^ phenotype in Ts-1 and Tsu-1. Plants displaying elevated shoot Na^+^ accumulation, compared to Col-0, were used to determine expression levels of At*HKT1* in shoot and root tissue, using quantitative real-time PCR. As previously described [15–17], expression of At*HKT1* in Col-0 is significantly higher in roots compared to shoots (Figure 4). In the shoot, no significant differences in expression levels were found between Col-0 and Ts-1 or Tsu-1. In the root, however, the level of At*HKT1* expression in Ts-1 and Tsu-1 is approximately 10-fold lower than in Col-0 (Figure 4).

**Figure 5:**
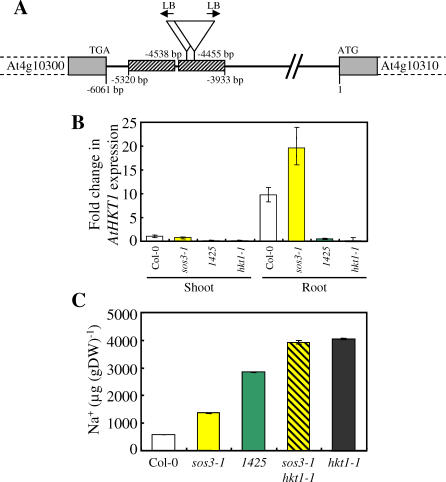
The Tandem Repeat Upstream of At*HKT1* Is Determinant for At*HKT1* Expression and Maintenance of Shoot Na^+^ Accumulation (A) Diagram of the T-DNA insertions in Line 1425. Represented are the two tandem repeats (hatched boxes), positions of the two T-DNA insertions (inverted triangles), and the arrows indicating the orientation of the left border (LB) for each T-DNA insertion. Numbering is based on the A of the At4g10310 start codon ATG as +1. The diagram is not drawn to scale. (B) At*HKT1* expression using quantitative real-time PCR in Col-0, *sos3–1* (Col-0 *gl1*), Line 1425, and *hkt1–1*. RNA was isolated from shoot and root of 6-wk-old plants grown in soil under short-day conditions. For normalization across samples, the expression of the *Actin 1* gene was used, and relative fold induction was calculated in comparison to At*HKT1* expression in Col-0 shoot using the ΔΔ*C*_t_ method. Presented data are the mean of at least three biological replicates, and the error bars represent ±SD. (C) Na^+^ accumulation in shoot tissue of Col-0, *sos3–1* (Col-0 *gl1*), Line 1425, *sos3–1 hkt1–1,* and *hkt1–1* grown for 6 wk in soil under short-day conditions (same plants used for quantitative real-time PCR above). Presented data are the mean ± SE (*n* = 12).

An *Arabidopsis* mutant (Line 1425), in the Col-0 *gl1 sos3–1* background, previously identified as a weak suppressor of *sos3–1* Na^+^ sensitivity (A. Rus and P. M. Hasegawa, unpublished data), was found to contain a T-DNA insertion within the tandem repeat region localized 5,320 to 3,933 bp upstream of the At*HKT1* start codon (Figure 5A), where Ts-1 and Tsu-1 also have a 725- and 687-bp deletion, respectively (Figure S2B). We observed that in Line 1425, expression of At*HKT1* in both roots and shoots is drastically reduced to levels comparable to *hkt1–1* (Figure 5B). Furthermore, disruption of this tandem repeat, and elimination of At*HKT1* expression in Line 1425, leads to elevated shoot Na^+^ compared to both Col-0 and *sos3–1* (Figure 5C), and this phenocopies the elevated shoot Na^+^ levels observed in both *hkt1–1* and the *sos3–1 hkt1–1* double mutant (Figure 5C). Such evidence strongly implicates the approximately 700-bp deletion in the tandem repeat upstream of At*HKT1* in both Ts-1 and Tsu-1 as being involved in the reduced root expression of At*HKT1* in these accessions.

### Grafting Establishes that HKT1 Function in Roots Is Critical for Regulation of Shoot Na^+^

**Figure 6:**
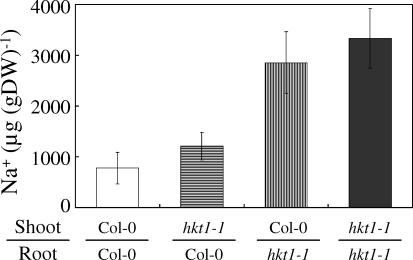
Root At*HKT1* Expression Is Determinant for Shoot Na^+^ Accumulation Na^+^ contents were analyzed in shoots of self-grafted Col-0 plants (*n* = 3), *hkt1–1* shoot grafted onto Col-0 roots (*n* = 14), Col-0 shoot grafted onto *hkt1–1* root (*n* = 9), and self-grafted *hkt1–1* plants (*n* = 3). Presented data are the mean ± SE.

The elevated shoot Na^+^ of Ts-1 and Tsu-1 that maps to the At*HKT1* locus is associated with the loss of At*HKT1* expression in roots. To address the contribution of *HKT1* expression in the root to the control of Na^+^ accumulation in the shoot, we performed reciprocal grafting experiments (Figure 6). Shoots from *hkt1–1* grafted onto Col-0 roots show no significant difference in shoot Na^+^ compared to Col-0 self-grafted controls, while Col-0 shoots grafted onto *hkt1–1* roots accumulated Na^+^ levels similar to those observed in self-grafted *hkt1–1* plants (Figure 6). This is further evidence that At*HKT1* expression in the root is the primary site for HKT1′s role in regulating shoot Na^+^.

### *HKT1* Allele in Ts-1 and Tsu-1 Is Associated with Enhanced NaCl Tolerance

**Figure 7:**
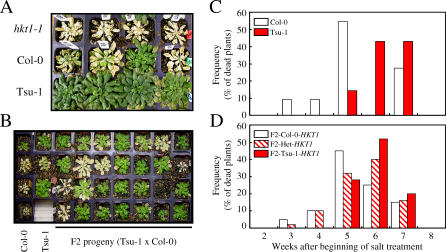
Contribution of Tsu-1 *HKT1* Allele to Survival Under NaCl Stress (A) Relative NaCl tolerance of *hkt1–1*, Col-0, and Tsu-1. Five-week-old *hkt1–1*, Col-0, and Tsu-1 plants were treated biweekly with 100 mM NaCl. Picture was taken 6 wk after beginning of the salt treatment. (B) NaCl tolerance of F2 plants from a Tsu-1 × Col-0 cross. Picture of one of four plant growth trays used in the experiment. Each tray includes plants from each parent (Col-0 and Tsu-1) as well as F2 plants. (C) Distribution of the parental lines Col-0 (*n* = 11) and Tsu-1 (*n* = 14) recorded dead each week after beginning of the salt treatment. Presented data are percentage of plants dead each week after beginning of salt treatment. (D) Distribution of F2 plants (Tsu-1 × Col-0, *n* = 95) genotyped as Col-0 homozygous or Tsu-1 homozygous or heterozygous for the *HKT1* allele and recorded dead each week after beginning of the salt treatment. Presented data are percentage of plants dead each week after beginning of salt treatment.

At*HKT1* has been implicated in *Arabidopsis* tolerance to NaCl stress. The *hkt1* null mutant is sensitive to elevated NaCl as a result of Na^+^ overaccumulation in the shoot [Figure 7A; 17–19]. Under unstressed conditions, we have established that the weaker At*HKT1* allele in Ts-1 and Tsu-1 is responsible for the elevated Na^+^ content in the shoot of these two accessions, similar to that observed in the *hkt1–1* null mutant. Under high NaCl concentrations (50 and 100 mM NaCl), both Ts-1 and Tsu-1 have higher levels of Na^+^ in the shoot compared to Col-0, in all leaves at different stages of development, including the youngest ones not fully developed and localized close to the meristem (stage 1; Figure S1B and S1C). However, this elevated shoot Na does not cause increased NaCl sensitivity in Tsu-1 (Figure 7A). Given the connection between At*HKT1* expression and NaCl sensitivity, we decided to evaluate the involvement of this novel allele of At*HKT1* in NaCl tolerance by screening F2 plants from a cross between Tsu-1 × Col-0 for their capacity to survive high levels of NaCl (Figure 7). The genotyping of the F2 population from Tsu-1 × Col-0 identified 25 plants homozygous for Tsu-1-*HKT1* and 50 plants heterozygous and 20 plants homozygous for the Col-0-*HKT1* allele, following the expected 1:2:1 segregation ratio. The plants homozygous or heterozygous for the Tsu-1-*HKT1* allele were able to survive longer in the presence of 100 mM NaCl compared to plants that were homozygous for the Col-0-*HKT1* allele (Figure 7). Therefore, although this new At*HKT1* allele contributes to an increased Na^+^ accumulation in the shoot, it does not confer NaCl sensitivity but, on the contrary, appears to co-segregate with enhanced NaCl tolerance. Although currently we cannot eliminate the possibility that a second unknown gene is conferring the enhanced NaCl tolerance observed in Tsu-1, and due to a small genetic distance between At*HKT1* and this second gene they segregate together in the F2 population studied. Further transgenic studies are required to discern this.

## Discussion

We have used natural variation in *Arabidopsis* to successfully identify a new At*HKT1* allele responsible for elevated Na^+^ accumulation in the shoots, compared to Col-0. We propose the reduced expression of At*HKT1* in roots as the cause of this enhanced Na^+^ accumulation and provide evidence which points to a deletion in a tandem repeat upstream of At*HKT1* as responsible for this differential regulation of At*HKT1* expression. Identification of At*HKT1* as a candidate gene was possible based on the mapping position obtained from our DNA microarray-based BSA analysis, combined with the known phenotype of *hkt1*. However, the difficulty arises in providing significant proof that At*HKT1* is the causal gene driving elevated shoot Na^+^. To achieve this, we have combined several lines of evidence as suggested by Weigel and Nordborg [23]. First, DNA polymorphisms allow us to distinguish between the At*HKT1* allele in Col-0 and the At*HKT1* allele found in both Ts-1 and Tsu-1. Second, the deficiency complementation test indicates that the new At*HKT1* allele found in Ts-1 and Tsu-1 is responsible for the Na^+^ accumulation in the shoot. Third, At*HKT1* shows a different expression pattern in Ts-1 and Tsu-1 compared to Col-0. Fourth, the characterization of this new At*HKT1* allele was performed in two independent genetic backgrounds, Ts-1 and Tsu-1, with the same results.

High-throughput elemental profiling of *Arabidopsis* mutagenized lines has proved to be very successful in identifying mutants with altered ionomes, some of which are caused by variation at a single locus [1,3]. As presented here, we have also found, using high-throughput elemental profiling coupled with genetics and DNA microarray-based mapping techniques, that the higher Na^+^ levels found in the shoots of Ts-1 and Tsu-1, two different *Arabidopsis* accessions, are due to natural variation at the At*HKT1* locus. AtHKT1 has been shown to transport Na^+^ and, based on studies involving the *hkt1* null mutants, has been implicated in Na^+^ distribution throughout the plant [15,17,18].

The concentrations of Na^+^ found in the shoots of Ts-1 and Tsu-1 are intermediate between the *hkt1–1* null mutant and Col-0 (Figures 3 and S1). This, coupled with the moderate complementation of the high Na^+^ phenotype of *hkt1–1* observed in the F1 plants from an *hkt1–1* × Ts-1 or Tsu-1 cross, suggests that the At*HKT1* allele in these accessions is likely not a null, but rather a weak, allele (Figure 3), although a genetic background effect cannot be excluded. Several amino acid changes occur in HKT1 from Ts-1 and Tsu-1; however, no nonsense mutation or frameshifts were found in the coding region (Figure S2A). Interestingly, deletions upstream of At*HKT1* are present in both Ts-1 and Tsu-1, and analysis of At*HKT1* expression revealed it to be dramatically reduced in roots of both Ts-1 and Tsu-1 but unaltered in shoots, compared to Col-0 (Figure 4). Such changes in expression levels are likely to be responsible for the accumulation of Na^+^ in the shoots of Ts-1 and Tsu-1.

Previously, a suppressor of the NaCl sensitivity of *sos3–1* (Col-0 *gl1*) called Line 1425 (A. Rus and P. M. Hasegawa, unpublished data) was identified as having a T-DNA insertion within the tandem repeat region upstream of At*HKT1* (Figure 5A), the same region that contains an approximately 700-bp deletion in both Ts-1 and Tsu-1 (Figure S2B). The reduction in At*HKT1* expression in Line 1425, which is associated with elevated shoot Na^+^ (Figure 5C), supports our hypothesis that the deletion in the tandem repeat region between 5,320 to 3,933 bp upstream of At*HKT1* is responsible for both the reduced expression of At*HKT1* in roots and elevated shoot Na^+^ observed in both Ts-1 and Tsu-1. Based on our sequence analysis and previous promoter-GUS reporter gene analysis [20], this tandem repeat region is well upstream of the promoter of At*HKT1*. However, further analysis of that region in the *Arabidopsis* mpss database ([24]; http://mpss.dbi.udel.edu/at) revealed the presence of a dense cluster of small RNAs from the unannotated tandem repeat upstream of At*HKT1*. We hypothesize that because this tandem repeat region appears to be involved in positively regulating expression of At*HKT1,* siRNAs or miRNAs produced from this region may be involved in interfering with a system that negatively regulates At*HKT1* expression. However, confirmation of this model requires further experimentation.

The availability of the natural variant of At*HKT1* in Ts-1 and Tsu-1, in which At*HKT1* expression is altered specifically in roots, supports a model for HKT1 function in which the transporter plays a role in controlling Na^+^ accumulation in shoots by regulating, at the root level, the transport of Na^+^ in the xylem transpiration stream to the shoot. We propose that lower expression of At*HKT1* in the roots results in reduced Na^+^ retrieval from the root xylem, leading to the elevated Na^+^ observed in Ts-1 and Tsu-1 shoots. Such a model is strongly supported by our grafting experiments which clearly demonstrate that activity of HKT1 in the roots is the major site of HKT1 function in regulating Na^+^ accumulation in shoots (Figure 6). These grafting experiments also demonstrate that the specific loss of HKT1 function in shoots has no significant effect on shoot accumulation of Na^+^ (Figure 6). This evidence refines the existing model which proposes that HKT1 also plays a significant role in retrieving Na^+^ from shoots and exporting Na^+^ from the shoot to root via the phloem [17,20], for which we find no evidence.

The *Arabidopsis* Na^+^ transporter HKT1 plays an important role in the shoot Na^+^ avoidance strategy of *Arabidopsis,* and because the new At*HKT1* allele reported here results in higher accumulation of Na^+^ in shoots (Figures 1, 3, and S1), we would expect plants containing this allele of At*HKT1* to be more sensitive to NaCl stress, as compared to Col-0. However, we observed this not to be the case. A comparison of the Col-0 and Tsu-1 ability to survive treatment with Na^+^ clearly shows that Tsu-1 survives longer than Col-0 (Figure 7). Furthermore, F2 plants from a cross between Col-1 × Tsu-1, either homozygous or heterozygous for the At*HKT1* allele from Tsu-1, were more NaCl tolerant than plants homozygous for the Col-0 At*HKT1* allele (Figure 7D). Such cosegregation of the Tsu-1 At*HKT1* allele with increased NaCl tolerance is consistent with this new allele of At*HKT1* being involved in the elevated tolerate to NaCl observed in Tsu-1. This evidence also confirms that in *Arabidopsis,* reduced shoot accumulation of Na^+^ is not necessary for enhanced salinity tolerance [25].

Both the Ts-1 and Tsu-1 accessions were collected from geographically (coastal regions of Spain and Japan) and genetically [21] distinct populations. However, both populations contain this new At*HKT1* allele, which makes it tempting to speculate that this allele allows these two populations to thrive in coastal regions where there might be enrichment of NaCl in the soil due to exposure to seawater. However, an extensive haplotype analyses will be required to identify if this new allele of At*HKT1* has been under recent directional selection before we can make any conclusive statements about its adaptive significance.

## Materials and Methods

### Plant materials.

A. thaliana Col-0, Ts-1, Tsu-1, and other accessions reported in this paper were obtained from the *Arabidopsis* Stock Center at the *Arabidopsis* Biological Resource Center. The *hkt1–1* mutant [19] was from our own seed stock. For the genetic analysis, F1 and F2 plants from different crosses are as specified throughout the paper.

### General plant growth conditions.

Plants used for elemental profiling by ICP-MS analysis were grown under unstressed conditions in a controlled environment, 8 h light:16 h dark (90 μmol • m^−2^ • s^−1^ light intensity) and 19 to 22 °C [1]. Briefly, seeds were sown onto moist soil (Sunshine Mix LB2; Carl Brehob & Son, Indianapolis, Indiana, United States) with various elements added at subtoxic concentrations (As, Cd, Co, Li, Ni, Pb, and Se [1]) and stratified at 4 °C for 3 d. Plants were bottom-watered twice per week with 0.25× Hoagland solution in which iron was replaced with 10 μM Fe-HBED [*N,N*′-di(2-hydroxybenzyl)ethylenediamine-*N,N*′-diacetic acid monohydrochloride hydrate; Strem Chemicals, Inc., http://www.strem.com). For sodium analysis after 42 d, plants were nondestructively sampled by removing one or two leaves. The plant material was rinsed with 18 MΩ water and placed into Pyrex digestion tubes.

### Screening of the F2 population from Tsu-1 × Col-0 for survival of NaCl stress.

The evaluation of the F2 population from the Tsu-1 × Col-0 cross in response to salt stress was realized in a growth chamber under controlled environmental conditions, 8 h light:16 h dark (150 μmol • m^−2^ • s^−1^ light intensity), 21 °C:19 °C (day/night) and 60% relative humidity. After stratification, F2 seeds as well as seeds from each one of the parents were sown onto moist soil (Scotts Potting Medium; Scotts-Sierra Horticultural Products Company, Marysville, Ohio, United States). About 2 wk after germination, seedlings were thinned to leave one plant per individual cell. Plants were bottom-watered twice per week with 0.5× Murashige and Skoog (MS) Macro- and Micronutrients (Caisson Laboratories, Inc., Rexburg, Idaho, United States). One month after the seeds were sewn, NaCl was added to the watering solution increasingly from 50 to 100 mM NaCl and maintained at that level until all the plants were recorded dead.

### Na^+^ accumulation in plants exposed to increasing concentrations of NaCl in soil.

Plants from Col-0, *hkt1–1,* Ts-1, and Tsu-1 used to evaluate Na^+^ accumulation in the shoot in response to NaCl stress were grown under controlled growth conditions (as described above in “General plant growth conditions”) and treated with NaCl for 2 wk. NaCl treatment was started 1 mo after the seeds were sewn. Plants were watered twice weekly without or with NaCl. The NaCl treatments included plants watered with 50 mM NaCl and plants watered once with 50 mM, once with 75 mM, and twice with 100 mM NaCl. Two weeks after the beginning of the salt treatments, plants were harvested and analyzed by ICP-MS. Na^+^ was analyzed in leaves of different age throughout the shoot. The leaves were numbered from the meristem toward the outer leaf ring of the rosette. Four samples (indicated as Nos. 1, 2, 3, and 4 in the graph) were harvested per plant, with No. 1 containing leaves Nos. 4 and 5 and therefore being the youngest leaves, No. 2 containing leaf No. 8, No. 3 containing leaf No. 11, and No. 4 containing leaf No. 14 and being the oldest.

### Tissue Na^+^ quantification.

Approximately 3 mg of dry weight of each plant was sampled into Pyrex tubes (16 × 100 mm) after drying at 92 °C for 20 h. After cooling, seven of approximately 100 samples from each sample set were weighed. All samples were digested with 0.7 ml of concentrated nitric acid (OmniTrace; VWR Scientific Products; http://www.vwr.com) and diluted to 6.0 ml with 18 MΩ water. Elemental analysis was performed with an ICP-MS (Elan DRCe; PerkinElmer, http://www.perkinelmer.com) for Li, B, Na, Mg, P, K, Ca, Mn, Fe, Co, Ni, Cu, Zn, As, Se, Mo, and Cd. All samples were normalized to calculated weights, as determined with an iterative algorithm using the best-measured elements, the weights of the seven weighed samples, and the solution concentrations, implemented in Microsoft Excel (http://www.microsoft.com) [1].

### DNA microarray-based BSA.

DNA microarray-based BSA was realized as previously described [12,13]. Briefly, SFPs were identified between Col-0 and Ts-1 and Tsu-1 by hybridizing labeled DNA from each one of the accessions to Affymetrix ATH1 microarrays and comparing them to Col-0 hybridizations downloaded from http://www.naturalvariation.org/xam. Two genomic DNA pools from an F2 population of a cross between Ts-1 or Tsu-1 and Col-0 were created and hybridized to arrays. Each one of the pools contained plants with either levels of Na^+^ similar to Col-0 (“control” pool) or high Na^+^ levels similar to Ts-1 or Tsu-1 (“high Na^+”^ pool). At loci unlinked to the ionomic phenotype of interest, the pools should have equivalent amounts of each genotype, and the hybridization signal at each SFP should be intermediate between the two parent accessions, for an average difference between the two DNA microarrays of zero. At linked loci, the difference between the two DNA microarrays should be approximately two-thirds the difference between the parent accessions. By smoothing the signal across multiple SFPs, the noise is reduced and the peak of the differences in hybridization signal will correspond to the chromosomal region of the loci controlling the mapped trait. Raw hybridization data (.CEL files) for each probe on the ATH1 DNA microarrays used in these experiments have been submitted to both Natrualvariation.org (http://www.naturalvariation.org) and Gene Expression Omnibus (http://www.ncbi.nlm.nih.gov/geo) for public distribution.

### Sequencing of the At*HKT1* allele from Ts-1 and Tsu-1.

To sequence the At*HKT1* gene and corresponding promoter from Ts-1 (CS1552) and Tsu-1 (CS1640), synthetic oligonucleotide primers were designed to produce overlapping amplified PCR products from 5,456 bp upstream of the At*HKT1* start codon to 386 bp downstream of the At*HKT1* stop codon, corresponding to positions 32,115 and 41,615, respectively of the BAC clone F24G24 in Chromosome IV.

Table S1 provides a summary of the pairs of primers used and the size of the PCR products expected for Col-0 (based on published sequence) and obtained for Ts-1 and Tsu-1. The PCR products obtained for each one of the primer pairs were cloned using the pGEM-T Easy Vector System I according to the manufacturer's instructions (Promega, http://www.promega.com). After transformation of electrocompetent Escherichia coli cells strain DH5α and selection of transformed colonies using ampicillin, positive clones were confirmed by PCR. The plasmids containing the inserted PCR product were extracted and used to carry out the sequencing reaction in both directions using Big Dye terminator v 3.0 according to the manufacturer's instructions (Applied Biosystems, http://www.appliedbiosystems.com). The sequencing products were then analyzed at the sequencing center (Purdue University), and sequences were compared to the corresponding Col-0 sequence from the *Arabidopsis* database.

### Quantitative real-time PCR.

Plants were first analyzed by ICP-MS and further used to determine the At*HKT1* transcript levels. Shoot and root from plants grown under short-day conditions (as described above in “General plant growth conditions”) were separated and rinsed thoroughly with deionized water to remove any soil contamination. The samples were then frozen in liquid nitrogen and stored at −80 °C until extraction. Total RNA was extracted from each one of the samples using the Qiagen RNeasy Plant Mini Kit (http://www.qiagen.com), and DNase digestion was performed during the extraction procedure according to the manufacturer's instructions. Two micrograms of total RNA was used as a template to synthesize first-strand cDNA with random hexamers using SuperScript II Reverse Transcriptase (Invitrogen Life Technologies, http://www.invitrogen.com). Quantitative real-time PCR was performed with the first strand cDNA as a template on a sequence detector system (ABI Prism 7000, Applied Biosystems). For normalization across samples, the expression of the *Actin 1* gene (At2g37620) was used with the following primers: CPRD66, 5′-TGG AAC TGG AAT GGT TAA GGC TG-3′ and CPRD67, TCT CCA GAG TCG AGC ACA ATA C-3′. For At*HKT1*, the following primers were used: HKT-RTF, 5′-TGG GAT CTT ATA ATT CGG ACA GTT C-3′ and HKT-RTR, 5′-GAT AAG ACC CTC GCG ATA ATC AGT-3′. For each sample, the average value from triplicate real-time PCRs was used to evaluate the transcript abundance, and the relative fold induction relative to At*HKT1* expression in Col-0 shoots was calculated based on the ΔΔC_t_ method.

### Grafting of *Arabidopsis*.

Seedlings to be grafted were germinated on 100 × 15-mm plates containing 0.5× MS Macro- and Micronutrients, 0.5× MS Vitamins (Caisson Laboratories, Inc.)*,* 3 mg/L Benomyl [methyl 1-(butylcarbamoyl)-2-benzimidazolecarbamate; Sigma, http://www.signaaldrich.com), 0.04 mg/L BA (6-benzylaminopurine; Sigma), 0.02 mg/L IAA (indole acetic acid; Sigma), and 12 g/L agar. Hormone treatment was found to greatly improve grafting efficiency due to at least three factors: enhanced callusing at the graft union, retardation of shoot growth which maintained contact at the graft union, and an approximately 90% reduction in adventitious root formation from the graft union. Benomyl virtually eliminated fungal contamination [26]. Plates containing the stratified seeds were placed vertically under controlled environmental conditions (16 h light:8 h dark and 25 °C). Five-day-old seedlings were grafted on the plate by the 90-degree blunt end technique with a 15-degree Stab Knife (Fine Scientific Tools, North Vancouver, British Columbia, Canada) without collars [27]. The grafted seedlings remained on the plate for an addition 5 d to allow the formation of the graft union. Successfully unified seedlings were transplanted directly to soil (as described above in “General plant growth conditions”). Plants were harvested for Na^+^ analysis 4 wk after transfer to soil. Postharvest analysis of graft unions was performed under the stereoscope to identify any adventitious root formation from grafted individuals. Individuals with adventitious roots emerging at or above the graft union were eliminated from subsequent analyses.

## Supporting Information

Figure S1 Higher Na^+^ Accumulation in Ts-1 and Tsu-1 Under Unstressed and NaCl Stress Conditions Na^+^ contents were analyzed in leaves at four different stages of development (as described in Materials and Methods) across the shoot of 44-d-old Col-0 (circles), Ts-1 (triangles), Tsu-1 (squares), and *hkt1–1* (diamonds) plants grown in soil under short-day conditions and treated for 17 d with (A) no NaCl addition, (B) 50 mM, or (C) 100 mM NaCl. Presented data are the mean ± SE.
(314 KB PPT)

Figure S2 Alignment of At*HKT1* cDNA and promoter sequences from Col-0, Ts-1, and Tsu-1(A) At*HKT1* cDNA sequences.(B) At*HKT1* promoter sequences. (85 KB DOC)

Table S1 List of Primer Pairs Used to Sequence the At*HKT1* Gene and PCR Product Size Expected for Col-0 and Obtained for Ts-1 and Tsu-1 (42 KB DOC)

### Accession Numbers

The *Arabidopsis* Biological Resource Center (ABRC) (http://www.biosci.ohio-state.edu/pcmb/Facilities/abrc/abrchome.htm12) accessions used in this paper were Col-0 (CS6000), Cvi-0 (CS1096), Est-1 (CS1150), Kas-2 (CS1264), Mrk-0 (CS1374), Mt-0 (CS1380), Se-0 (CS1502), Ts-1 (CS1552), Van-0 (CS1584), Nd-1 (CS1636), Tsu-1 (CS1640), and L*er*-2 (CS8581).

## Abbreviations

BSA: bulk segregant analysis
SFP: single feature polymorphism
SNP: single nucleotide polymorphism
Ts-1: *Arabidopsis* accessions collected from Tossa del Mar, Spain
Tsu-1: *Arabidopsis* accessions collected from Tsu, Japan
