# Supplementary material for: Natural Variants of AtHKT1 Enhance Na+ Accumulation in Two Wild Populations of Arabidopsis
Source: PLoS Genet. 2006 Dec 1;2(12):e210. doi: 10.1371/journal.pgen.0020210 (PMC1665649; doi:10.1371/journal.pgen.0020210)
Supplement: Supplementary file 1 [file pgen.0020210.sg002.doc]

Figure S2. Alignment of At*HKT1* cDNA (A) and promoter (B) sequences from Col-0, Ts-1 (CS 1552) and Tsu-1 (CS 1640).

A. Alignment of At*HKT1* cDNA sequences from Col-0, Ts-1 and Tsu-1.

```
1                                                                             80
 Col-0        (1) ATGGACAGAGTGGTGGCAAAAATAGCAAAAATCCGTTCGCAGCTTACTAAATTACGTTCACTATTCTTCCTTTACTTCAT
 Ts-1         (1) ATGGACATAGTGGTGGCAAAAATAGCAAACATTCGTTCGCAGCTTACTAAATTACGTTCTCTATTCTTCCTTTACTTCAT
 Tsu-1        (1) ATGGACATAGTGGTGGCAAAAATAGCAAACATTCGTTCGCAGCTTACTAAATTACGTTCTCTATTCTTCCTTTACTTCAT

                  81                                                                           160
 Col-0       (81) CTACTTCTTGTTCTTCTCCTTTTTAGGGTTTTTGGCACTCAAGATCACAAAGCCAAGAACCACTTCACGTCCTCATGACT
 Ts-1        (81) CTACTTCTTGTTCTTCTCCTTTTTAGGGTTTTTGGCACTCAAGATCACAAAGCCAAGAACCACTTCACGTCCTCATGACT
 Tsu-1       (81) CTACTTCTTGTTCTTCTCCTTTTTAGGGTTTTTGGCACTCAAGATCACAAAGCCAAGAACCACTTCACGTCCTCATGACT

                  161                                                                          240
 Col-0      (161) TTGACCTTTTCTTCACTTCTGTCTCTGCCATCACCGTCTCTTCCATGTCTACCGTCGACATGGAAGTCTTCTCCAACACC
 Ts-1       (161) TTGACCTTTTCTTCACTTCTGTCTCTGCCATAACCCTCTCTTCCATGTCTACCGTCGACATGGAAGTCTTCTCCAACACC
 Tsu-1      (161) TTGACCTTTTCTTCACTTCTGTCTCTGCCATAACCCTCTCTTCCATGTCTACCGTCGACATGGAAGTCTTCTCCAACACC

                  241                                                                          320
 Col-0      (241) CAACTTATCTTCCTCACTATCCTCATGTTCCTCGGTGGCGAAATCTTCACCTCCTTTCTCAACCTCTACGTCTCCTATTT
 Ts-1       (241) CAACTTATCTTCCTCACTATCCTCATGTTCCTTGGTGGCGAAATCTTCACCTCCTTTCTCAACCTCTATGTCTCCTATTT
 Tsu-1      (241) CAACTTATCTTCCTCACTATCCTCATGTTCCTTGGTGGCGAAATCTTCACCTCCTTTCTCAACCTCTATGTCTCCTATTT

                  321                                                                          400
 Col-0      (321) CACCAAGTTCGTCTTCCCTCATAACAAGATTAGACATATTTTGGGATCTTATAATTCGGACAGTTCCATCGAGGATCGCT
 Ts-1       (321) CACCAAGTTCGTCTTCCCTCATAACAAGATTAGACATATTTTGGGATCTTATAATTCGGACAGTTCCATCGAGGATCGCT
 Tsu-1      (321) CACCAAGTTCGTCTTCCCTCATAACAAGATTAGACATATTTTGGGATCTTATAATTCGGACAGTTCCATCGAGGATCGCT

                  401                                                                          480
 Col-0      (401) GTGACGTTGAGACTGTTACTGATTATCGCGAGGGTCTTATCAAGATCGATGAAAGGGCATCTAAGTGCTTGTACTCGGTG
 Ts-1       (401) ATGACGTTGAGACTGTTACTGATTATCGCGAGGGTCTTATCAAGATCGATGAAAGGGCATCTAAGTGCTTGTACTCGGTG
 Tsu-1      (401) ATGACGTTGAGACTGTTACTGATTATCGCGAGGGTCTTATCAAGATCGATGAAAGGGCATCTAAGTGCTTGTACTCGGTG

                  481                                                                          560
 Col-0      (481) GTTCTTAGTTACCATCTTGTTACTAACCTAGTTGGCTCTGTGTTGCTTCTTGTGTACGTAAATTTTGTTAAAACGGCGAG
 Ts-1       (481) GTTCTTAGTTACCATCTTGTTACTAACCTAGTTGGCTCTGTGTTGCTTCTTGTGTACGTAAATTTTGTTAAAACGGCGAG
 Tsu-1      (481) GTTCTTAGTTACCATCTTGTTACTAACCTAGTTGGCTCTGTGTTGCTTCTTGTGTACGTAAATTTTGTTAAAACGGCGAG

                  561                                                                          640
 Col-0      (561) AGATGTTCTTAGTTCCAAAGAAATCTCACCTCTCACTTTCTCCGTCTTCACAACTGTTTCCACGTTTGCAAACTGCGGAT
 Ts-1       (561) AGATGTTCTTAGTTCCAAAGAAATCTCACCTCTCACTTTCTCCGTCTTCACAACAGTTTCCACGTTTGCAAACTGCGGAT
 Tsu-1      (561) AGATGTTCTTAGTTCCAAAGAAATCTCACCTCTCACTTTCTCCGTCTTCACAACAGTTTCCACGTTTGCAAACTGCGGAT

                  641                                                                          720
 Col-0      (641) TTGTCCCCACGAATGAGAACATGATCATCTTTCGCAAGAACTCTGGTCTCATCTGGCTCCTAATCCCTCAAGTACTGATG
 Ts-1       (641) TTGTCCCTACGAATGAGAATATGATCATCTTTCGCAAGAACTCTGGTCTCATCTGGCTCCTAATCCCTCAAGTACTGATG
 Tsu-1      (641) TTGTCCCTACGAATGAGAATATGATCATCTTTCGCAAGAACTCTGGTCTCATCTGGCTCCTAATCCCTCAAGTACTGATG

                  721                                                                          800
 Col-0      (721) GGAAACACTTTGTTCCCTTGCTTCTTGGTTTTGCTCATATGGGGACTTTATAAGATCACAAAGCGTGACGAGTATGGTTA
 Ts-1       (721) GGAAACACTTTGTTCCCTTGCTTCTTGGTTTTGCTCATATGGGGACTTTATAAGATCACAAAGCGTGACGAGTATGGTTA
 Tsu-1      (721) GGAAACACTTTGTTCCCTTGCTTCTTGGTTTTGCTCATATGGGGACTTTATAAGATCACAAAGCGTGACGAGTATGGTTA

                  801                                                                          880
 Col-0      (801) CATTCTCAAGAACCACAATAAGATGGGATACTCTCATCTACTCTCGGTTCGTCTATGTGTTCTTCTTGGAGTGACGGTGC
 Ts-1       (801) CATTCTCAAGAACCACAATAAGATGGGATACTCTCATCTACTCTCGGTTCGTCTATGTGTTCTTCTTGGAGTGACGGTGC
 Tsu-1      (801) CATTCTCAAGAACCACAATAAGATGGGATACTCTCATCTACTCTCGGTTCGTCTATGTGTTCTTCTTGGAGTGACGGTGC

                  881                                                                          960
 Col-0      (881) TAGGGTTTCTGATAATACAGCTTCTTTTCTTCTGCGCCTTTGAATGGACCTCTGAGTCTCTAGAAGGAATGAGTTCGTAC
 Ts-1       (881) TAGGGTTTCTGATAATACAGCTTCTTTTCTTCTGCGCCTTTGAATGGACCTCTGAGTCTCTAGAAGGAATGAGTTCGTAC
 Tsu-1      (881) TAGGGTTTCTGATAATACAGCTTCTTTTCTTCTGCGCCTTTGAATGGACCTCTGAGTCTCTAGAAGGAATGAGTTCGTAC

                  961                                                                         1040
 Col-0      (961) GAGAAGTTGGTTGGATCGTTGTTTCAAGTGGTGAATTCGCGACACACCGGAGAAACTATAGTAGACCTCTCTACACTTTC
 Ts-1       (961) GAGAAGTTGGTTGGATCGTTGTTTCAAGTGGTGAATTCGCGACACACCGGAGAAACTATAGTAGACCTCTCTACACTTTC
 Tsu-1      (961) GAGAAGTTGGTTGGATCGTTGTTTCAAGTGGTGAATTCGCGACACACCGGAGAAACTATAGTAGACCTCTCTACACTTTC

                  1041                                                                        1120
 Col-0     (1041) CCCAGCTATCTTGGTACTCTTTATTCTTATGATGTATCTTCCTCCATACACTTTATTTATGCCGTTGACGGAACAAAAGA
 Ts-1      (1041) CCCAGCTATCTTGGTACTCTTTATTCTTATGATGTATCTTCCTCCATACACTTTATTTATGCCGTTGACGGAACAAAAGA
 Tsu-1     (1041) CCCAGCTATCTTGGTACTCTTTATTCTTATGATGTATCTTCCTCCATACACTTTATTTATGCCGTTGACGGAACAAAAGA


                  1121                                                                        1200
 Col-0     (1121) CGATAGAGAAAGAAGGAGGAGATGATGATTCCGAAAATGGAAAGAAAGTTAAAAAGAGTGGACTCATCGTGTCACAACTT
 Ts-1      (1121) CGATAGAGAAAGAAGGAGGAGATGATGATTCCGGAAATGGAAAGAAAGTTAAAAAGAGTGGACTCATCGTGTCACAACTT
 Tsu-1     (1121) CGATAGAGAAAGAAGGAGGAGATGATGATTCCGGAAATGGAAAGAAAGTTAAAAAGAGTGGACTCATCGTGTCACAACTT

                  1201                                                                        1280
 Col-0     (1201) TCCTTTTTGACGATATGTATCTTTCTCATTTCAATCACCGAAAGGCAAAATCTACAACGTGATCCGATAAATTTCAACGT
 Ts-1      (1201) TCCTTTTTGACGATATGTATCTTTCTCATTTCAATCACCGAAAGGCAAAATCTACAACGTGATCCGATAAATTTCAACGT
 Tsu-1     (1201) TCCTTTTTGACGATATGTATCTTTCTCATTTCAATCACCGAAAGGCAAAATCTACAACGTGATCCGATAAATTTCAACGT

                  1281                                                                        1360
 Col-0     (1281) CCTTAACATCACTCTCGAAGTTATCAGTGCATATGGAAACGTTGGTTTCACTACCGGGTACAGCTGTGAACGGCGTGTGG
 Ts-1      (1281) CCTTAACATCACTCTCGAAGTTATCAGTGCATATGGAAACGTGGGTTTCACTACCGGGTACAGCTGTGAACGGCGTCTGG
  Tsu-1    (1281) CCTTAACATCACTCTCGAAGTTATCAGTGCATATGGAAACGTGGGTTTCACTACCGGGTACAGCTGTGAACGGCGTCTGG

                  1361                                                                        1440
 Col-0     (1361) ACATCAGCGATGGTGGCTGCAAAGACGCGAGTTATGGGTTTGCAGGACGATGGAGTCCAATGGGAAAATTCGTACTAATA
 Ts-1      (1361) ACATCAGCGATGGTGGCTGCAAAGACGCGAGTTATGGGTTTGCAGGACGATGGAGTCCAATGGGTAAATTGGTACTAATA
 Tsu-1     (1361) ACATCAGCGATGGTGGCTGCAAAGACGCGAGTTATGGGTTTGCAGGACGATGGAGTCCAATGGGTAAATTGGTACTAATA

                  1441                                                                        1520
 Col-0     (1441) ATAGTAATGTTTTATGGTAGGTTTAAGCAGTTCACAGCCAAATCTGGCCGCGCATGGATTCTTTACCCCTCGTCTTCCTA
 Ts-1      (1441) ATAGTGATGTTTTATGGTAGGTTTAAGCAGTTCACAGCCAAATCTGGCCGCGCATGGATACTTTACCCCTCGTCTTCCTA
 Tsu-1     (1441) ATAGTGATGTTTTATGGTAGGTTTAAGCAGTTCACAGCCAAATCTGGCCGCGCATGGATACTTTACCCCTCGTCTTCCTA

                  1521
 Col-0     (1521) A
 Ts-1      (1521) A
 Tsu-1     (1521) A
```

B. Alignment of At*HKT1* promoter sequences from Col-0, Ts-1 and Tsu-1.

```
                1                                                                                                                    120
    Col-0     (1) AGAAATCGCTCAACAAACTACTACGTACTAATCGACTAAGCTTCATTCTTGTGCAGCCTATTCCTAGCTACTCAATCACGTTGTCTAGTAACTAACCAGGCTAATTTGCAATGTTCATAG
     Ts-1     (1) AGAAATCGCTCAACAAACTACTACGTACTAATCGACTAAGCTCCATTCTTGTGCAGCCTATTCCTAGCTACTCAATCACGTTGTCTAGTAACTAACCAGGCTAATTTGCAATGTTCATAG
    Tsu-1     (1) AGAAATCGCTCAACAAACTACTACGTACTAATCGACTAAGCTCCATTCTTGTGCAGCCTATTCCTAGCTACTCAATCACGTTATCTAGTAACTAACCAGGCTAATTTGCAATGTTCATAG

                  121                                                                                                                  240
    Col-0   (121) GTATTTGAGTAAAGAACTCAGTTAAAAAGTTTCATCAAGCAATACTACAACAAAAACACATCAAGTCCGCATACACGTTCCACAATTCACGCCATCTTTGACCTATCATTTAGGTCATTG
     Ts-1   (121) GTATTTGAGTAAAGAACTCAGTTAAAAAGTTTCATCAAGCAATAATACAACAAAAACACATCAAGTCCGCATACACGTTCCACAATTCACGCCATCTTTGACCTATCATTTAGGTCATTG
    Tsu-1   (121) GTATTTGAGTAAAGAACTCAGTTAAAAAGTTTCATCAAGCAATAATACAACAAAAACACATCAAGTCCGCATACACGTTCCACAATTCACGCCATCTTTGACCTATCATTTAGGTCATTG

                  241                                                                                                                  360
    Col-0   (241) TGTTAATTACATCATTATCCATGGCCAACAATTCATAACACCAAAACCATCACAAACTTATTTCAAATCTATCATGCAACTAGGGTTTCAAAATTGTAAAATTTCGCAGGTGACCTTACT
     Ts-1   (241) TGTTAATTACATCATTATCCATGGCCAACAATTCATAGCACCAAAACCATCACAAACTTATTTCAAATCTATCATGCAACTAGGGTTTCAAAATTGTAAAATTTCGCAGGTGACCTTACT
    Tsu-1   (241) TGTTAATTACATCATTATCCATGGCCAACAATTCATAGCACCAAAAC----ACAAACTTATTTCAAATCTATCATGCAACTAGGGTTTCAAAATTGTAAAATTTCGCAGGTGACCTTACT

                  361                                                                                                                  480
    Col-0   (361) AGTAATAGGTCCGGCTGACATATATATATATATATATATATATATATATAAAGACATTAAAAGAGAGGTCACTTAAGTTAAAATGGAACTATCGACGAGTAAGTGACCTTTCGTCTTTTT
     Ts-1   (361) AGTAATAGGTCCGGCTGACACACATA----------------------------------------------------------------------------------------------
    Tsu-1   (357) AGTAATAGGTCCGGCTGACACACACATATATATATATATATATATATATA----------------------------------------------------------------------

                  481                                                                                                                  600
    Col-0   (481) TATTTTTCCATTAGTGTATAATGTAACTTTTACTTCATTAGTGTTACTTTTCCTCGATACTTGGTGGCTCTGAATAATATTATCTCTTAGAAGATCTCCGAAGTTGTCGTATAAAAATTC
     Ts-1   (387) ------------------------------------------------------------------------------------------------------------------------
    Tsu-1   (407) ------------------------------------------------------------------------------------------------------------------------

                  601                                                                                                                  720
    Col-0   (601) ATTTACAAACCTAGGTGGAGATAACATTTTAAAAGAATGGATTCTTTAGTGCTAAACGCATTTAAGAAAGATGAACATATTTTGTTCGTCCTCTATTGGCAACATCTTTATCAAGACTCG
     Ts-1   (387) ------------------------------------------------------------------------------------------------------------------------
    Tsu-1   (407) ------------------------------------------------------------------------------------------------------------------------

                  721                                                                                                                  840
    Col-0   (721) AATTTAATTCTCTTTAAAAAGAGATTTAAATATGTTAATGATTTAAAAGTGGCCATCTAGCAAGTGATTGATACATCACGATTTAAATATTATTATCAAGCAATAATATTTCATCTAGCA
     Ts-1   (387) ------------------------------------------------------------------------------------------------------------------------
    Tsu-1   (407) ------------------------------------------------------------------------------------------------------------------------

                  841                                                                                                                  960
    Col-0   (841) AGTGATTGATACTCAGTTAAAAAGTTTCATCAAGCAATAATACAACAAAAACACATCAAGTCCGCATACACGTTCCACAATTCACGCCATCTTTGACCTATCATTTAGGTCATTGTGTTA
     Ts-1   (387) ------------------------------------------------------------------------------------------------------------------------
```

Tsu-1	(407) ------------------------------------------------------------------------------------------------------------------------

```
                  961                                                                                                                 1080
    Col-0   (961) ATTACATTATTATCCATGGCCAACAATTCATAGCACCAAAACCATCACAAACTTATTTCAAATCTATCATGCAACTAGGGTTTCAAAATTGTAAAATTTCGCAGGTGACCTTACTAGTAA
     Ts-1   (387) ------------------------------------------------------------------------------------------------------------------------
    Tsu-1   (407) ------------------------------------------------------------------------------------------------------------------------

                  1081                                                                                                                1200
    Col-0  (1081) TAGGTCCGGCTGACACATATATATATAT----------ATATATATAAAAACACTAAAAGAGAGGTCACTTAAGTTAAAATGGTACTATCGACGAGTAAGTGACCTTTCGTCTTTTTTAT
     Ts-1   (387) -----------------------------------------TATATAAAGACACTAAAAGAGAGGTCACTTAAGTTAAAATGGTACTATCGACGAGTAAGTGACCTTTCGTCTTTTTTAT
    Tsu-1   (407) -----------------TATATATATATATATATATATATATATATAAAGACACTAAAAGAGAGGTCACTTAAGTTAAAATGGTACTATCGACGAGTAAGTGACCTTTCGTCTTTTTTAT

                  1201                                                                                                                1320
    Col-0  (1191) TTTTCCATTAGTGTATAATATAACTTTTACTTCATTAGTGTTACTTTTCCTCGATACTTAATGGCTCTGAATAATATTATCTCTTAGAAGATCTCCGAAGTTGTTGTATAAAAATTCATT
     Ts-1   (466) TTTTCCATTAGTGTATAATGTAACTTTTACTTCATTAGTGTTACTTTTCCTCGATACTTGGTGGCTCTGAATAATATTATCTCTTAGAAGATCTCCGAAGTTGTCGTATAAAAATTCATT
    Tsu-1   (510) TTTTCCATTAGTGTATAATGTAACTTT-ACTTCATTAGTGTTACTTTTCCTCGATACTTGGTGGCTCTGAATAATATTATCTCTTAGAAGATCTCCGAAGTTGTCGTATAAAAATTCATT

                  1321                                                                                                                1440
    Col-0  (1311) TACAAACTTAGGTGGAGATAACATTTTAAAAGAATGGATTCTTTAGTGCTAAACGCATTTAAGAAAGATGAACATATTTTGTTCGTCCTCTATTGGCAACATCTTTATCAAGACTCGAAT
     Ts-1   (586) TACAAACCTAGGTGGAGATAACATTTTAAAAGAATGGATTCTTTAGTGCTAAACGCATTTAAGAAAGATGAACATATTTTGTTCGTCCTCTATTGGCAACATCTTTATCAAGACTCGAAT
    Tsu-1   (629) --CAAACCTAGGTGGAGATAACATTTTAAAAGAATGGATTCTTTAGTGCTAAACGCATTTAAGAAAGATGAACATATTTTGTTCGTCCTCTATTGGCAACATCTTTATCAAGACTCGAAT

                  1441                                                                                                                1560
    Col-0  (1431) TTAATTCTCTTTAAAAAGAGATTTAAATATGTTAATGATTTAAAAGTGGCCATCTAGCAAGTGATTGATACATCACGATTTAAATATTATTATAATATCACTTCCTAGATTGTGTCCCTT
     Ts-1   (706) TTAATTCTCTTTAAATAGAGATTTAAATATGTTAATGATTTAAAAGTGGCCACCTAGCAAGTGATTGATACATCACGATTTAAATATTATTATAATATCACTTCCTAGATTGTGTCCCTT
    Tsu-1   (747) TTAATTCTCTTTAAATAGAGATTTAAATATGTTAATGATTTAAAAGTGGCCACCTAGCAAGTGATTGATACATCACGATTTAAATATTATTATAATATCACTTCCTAGATTGTGTCCCTT

                  1561                                                                                                                1680
    Col-0  (1551) TCCTAGTCCCTATCATACAATAGTTATCCTCGTGCACATAACACTCTTAAAAATACACACCATCTTGTAATTCAAATTTGATGTGTTCGTACATATTTTCAACCATTTGTTTCTATTTCA
     Ts-1   (826) TCCTAGTCCCTATCATACAATAGTTACCCTCGTGCACATAACACTCTTAAAAATACACACCATCTTGTAATTCAAATTTGATGTGTTCGTACATATTTTCAACCATTTGTTTCTATTTCA
    Tsu-1   (867) TCCTAGTCCCTATCATACAATAGTTACCCTCGTGCACATAACACTCTTAAAAATACACACCATCTTGTAATTCAAATTTGATGTGTTCGTACATATTTTCAACCATTTGTTTCTATTTCA

                  1681                                                                                                                1800
    Col-0  (1671) AATTCTTCTCGTAAAAATATGTCCATTTAACCAGAAGTGGATAGCTCGACCGGAAACATACTAATTTTTTTTT-AAATATCACATCGAGTGACTGCGGGTATAAGTAAAAATATTAAAGA
     Ts-1   (946) AATTCTTCTCGTAAAAATATGTCCATTTAACCAGAAGTGGATAGCTCGACCGGAAACATACTATTTTTTTTTTTAAATATCACATCGAGTGACTGCGGGTATAAGTAAAAATATTAAAGA
    Tsu-1   (987) AATTCTTCTCGTAAAAATATGTCCATTTAACCAGAAGTGGATAGCTCGACCGGAAACATACTATTTTTTTTTTTAAATATCACATCGAGTGACTGCGGGTATAAGTAAAAATATTAAAGA

                  1801                                                                                                                1920
    Col-0  (1790) CTAATCCGAACATAAAAAATTATTAGAGCATCCGCATTGCACATTTAGTTTAAATGTATTTCCACATAAATTTAATTCATAAAATAAGAAAATGGTCTTTAAATGAAAGATATCCTTCCA
     Ts-1  (1066) CTAATCCGAACATAAAAAATTATTAGAGCATCAGCATTGCACATTTAGTTTAAATGTATTTCCACATAAATTTAATTCATGAAATAAGAAAATGGTCTTTAAATGAAAGATATCCTTCCA
    Tsu-1  (1107) CTAATCCGAACATAAAAAATTATTAGAGCATCAGCATTGCACATTTAGTTTAAATGTATTTCCACATAAATTTAATTCATAAAATAAGAAAATGGTCTTTAAATGAAAGATATCCTTCCA

                  1921                                                                                                                2040
    Col-0  (1910) AAAATGTGTCAAAGAAGACATTTTGAAACACATTGAAAAATGGGTCTTCCATTTAAAAAGACTATTCATACTTTATTAATAATATTTATATGAGAATACACACAAAAAATATATGCGATG
     Ts-1  (1186) AAAATGTGTCAAAGAAGACATTTTGAAACACATTGAAAAATGGGTCTTCCATTTAAAGAGACTATTCATACTTTATTGATAATATTTATATGAGAATACACACAAAATATATATGCAATG
```

Tsu-1 (1227) AAAATGTGTCAAAGAAGACATTTTGAAACACATTGAAAAATGGGTCTTCCATTTAAAAAGACTATTCATACTTTATTGATAATATTTATATGAGAATACACACAAAATATATATGCAATG

```
                  2041                                                                                                                2160
    Col-0  (2030) TGGGTGGTCTTATGGGCTTTTGCCCGAAATCTAAGTATGGTAACTAAAAATAAGGGCATAAAAACATCCACATTGGTGTACACTCTTAAAAAAGTAGTTAGTATAATTTATTATAAAAGC
     Ts-1  (1306) TGGGTGGTCTTATGGGCTTTTGCCCGAAATCTAAGTATGGTAACTAAAAATAAGGGCATAAAAACATCCACATTGGTGTACACTCTTAAAAATGTAGTTAGTATAATTTATTATAAAAGC
    Tsu-1  (1347) TGGGTGGTCTTATGGGCTTTTGCCCGAAATCTAAGTATGGTAACTAAAAATAAGGGCATAAAAACATCCACATTGGTGTACACTCTTAAAAATGTAGTTAGTATAATTTATTATAAAAGC


                  2161                                                                                                                2280
    Col-0  (2150) TAATATTATTGGGTAATATTAGAAAATAATCATAGAATTGAAAAGAATGAAGTAAGTAATCATTGTCAGTTTGTATGTGAGTAAATAACCTTATGACGACATAAGAAAATACAAAAATGT
     Ts-1  (1426) TAATATTATTGGGTAATATTAGAAAATAACCATAGAATTGAAAAGAATGAAGTAAGTAACCATTGTCAGTTTGTATGTGAGTAAATAACCTTATGACGACATAAAAAAATACAAAAATGT
    Tsu-1  (1467) TAATATTATTGGGTAATATTAGAAAATAACCATAGAATTGAAAAGAATGAAGTAAGTAACCATTGTCAGTTTGTATGTGAGTAAATAACCTTATGACGACATAAAAAAATACAAAAATGT

                  2281                                                                                                                2400
    Col-0  (2270) TCTCGCAAGTTGAACCAAAAGCAAGAGTATGAGAATAGCATGATATTTAAAAAAAAAGTGACATTTT-------------ATAATGTGTTATGAGAAAAAATAGCATGTTGACAGAGAAA
     Ts-1  (1546) TCTCGCAAGTTGAACCAAAAGCAAGAGTATGAGAATAGCATGATATTTAAAAAAAAAGTGACAATGTGTTATGAGAATAAATAATGTGTTATATGAAAAAATAGCATGTTGACAGAGAAA
    Tsu-1  (1587) TCTCGCAAGTTGAACCAAAAGCAAGAGTATGAGAATAGCATGATATTTTAAAAAAAAGTGACAATGTGTTATGAGAATAAATAATGTGTTATATGAAAAAATAGCATGTTGACAGAGAAA

                  2401                                                                                                                2520
    Col-0  (2377) GATTTCCACTTATCACGTGTAATTCAAAAAAGTAGCATGGTACAAAAGAAAATAACATACAATTTAAAATGAAAATATAACGACAAAAATTCCAATAAAAAATCAAAGTCGAACAAAATA
     Ts-1  (1666) GATTTCCACTTGTCACGTGTAATTCAAAAAAGTAGCATGGTACAAAAGAAAATAACATACAATTTAAAATGAAAATATAACGACGAAAATTCCAATAAAAAATCAAAATCGAACAAAATA
    Tsu-1  (1707) GATTTCCACTTGTCACGTGTAATTCAAAAAAGTAGCATGGTACAAAAGAAAATAACATACAATTTAAAATGAAAATATAACGACGAAAATTCCAATAAAAAATCAAAATCGAACAAAATA

                  2521                                                                                                                2640
    Col-0  (2497) AATAGCACGATATTTATCAAAAAGTGACATTTTATAATGTGCTATGAGAACTAATAATTTGTTATATGAAAAAATAGCATGTTGTTTGAAAGAGATTTTTAATTATAACGTGTAATTCAC
     Ts-1  (1786) AATAGCACGATATTTATCAAAAAGTGACATTTTATAATGTGCTATGAGAACTAATAATTTGTTATATGAAAAAATAGCATGTTGTTTGAGAGAGATTTTTAATTATAACGTGTAATTCAC
    Tsu-1  (1827) AATAACATGACATTTATCAAAAAGTGACATTTTATAATGTGCTATGAGAACTAATAATTTGTTATATGAAAAAATAGCATGTTGTTTGAGAGAGATTTTTAATTATAACGTGTAATTCAC

                  2641                                                                                                                2760
    Col-0  (2617) AAAAGTAGCATGGTAAAAAAGAAAATAACATACAATTTAAAAAGAAAATATAACGACGGAGATTCCAAGAAAGAATCAAAATCAAACAAAATAAAATGAGAAATCTTCTGCAAATTGAGG
     Ts-1  (1906) AAAAGTAGCATGGTAAAAAAGAAAATAACATACAATTTAAAAAGAAAACATAACGACGGAGATTCCAAGAAAGAATCAAAATCAAACAAAATAAAATGAGAAATCTTCTGCAAATTGAGG
    Tsu-1  (1947) AAAAGTAGCATGGTAAAAAAGAAAATAACATACAATTTAAAAAGAAAACATAACGACGGAGATTCCAAGAAAGAATCAAAATCAAACAAAATAAAATGAGAAATCTTCTGCAAATTGAGG

                  2761                                                                                                                2880
    Col-0  (2737) GAGAGATGATCCGTCAATATAAATAACGTTTAGCAGCCTAGATTTATCGAAAAAGAGATCTGGTGGTTGTGATGGTGGAAGATGTGGTGGTTGTGGTGGTGGAAGAAAGAGAGATGAGTG
     Ts-1  (2026) GAGAGATGATCCGTCAATATAAATAACGTTTAGCAGCCTAGATTTATCGAAAAGGAGATCTGGTGGTTGTGATGGTGGAAGATGTGGTGGTTGTGGTGGTGGAAGAAAGAGAGATGAGTG
    Tsu-1  (2067) GAGAGATGATCCGTCAATATAAATAACGTTTAGCAGCCTAGATTTATCGAAAAAGAGATCTGGTGGTTGTGATGGTGGAAGATGTGGTGGTTGTGGTGGTGGATGAAAGAGAGATGAGTG

                  2881                                                                                                                3000
    Col-0  (2857) GTGGTTTGTTCGGGTTCATCAAAAGTTTTTCGCCATGAAATCATCTAAGTGTTCTATGTGATTTTGGAAAATAGCGTGTAATACATATAAAATTCGACAATGGGTAAAAAGGTAATTGCA
     Ts-1  (2146) GTGGTTTGTTCGGGTTCATCAAAAGTTTTTCGCCATGAAATCATCTAAGTGTTCTATGTGATTTTGGAAAATAGCGTGTAATACATATAAAATTCGATAATGGGTAAAAAGGTAATTGCA
    Tsu-1  (2187) GTGGTTTGTTCGGGTTCATCAAAAGTTTTTCGCCATGAAATCATCTAAGTGTTCTATGTGATTTTGGAAAATAGCGTGTAATACATATAAAATTCGATAATGTGTAAAAAGATAATTGCA

                  3001                                                                                                                3120
    Col-0  (2977) AGTGAAAAAAAAGAGTGTACTCTGACACCATAGCTACTTTGTTAATTCTTTTAAGATTCATAGTTATTTTGTGCATATTCTCAATATTATTTTAATTTTGAGGTATTCATTAACCATGTA
     Ts-1  (2266) AGTAAAAAAAAAGAGTGTACTCTGACACCATAGCTGCTTTGTTAATTCTTTTAAGATTCATAGTTATTTTGTGCATATTCTCAATATTATTTTAATTTTGAGGTATTCATTAACCATGTA
```

Tsu-1 (2307) AGTAAAAAAAAAGAGTGTACTCTGACACCATAGCT**G**CTTTGTTAATTCTTTTAAGATTCATAGTTATTTTGTGCATATTCTCAATATTATTTTAATTTTGAGGTATTCATTAACCATGTA

```
                  3121                                                                                                                3240
    Col-0  (3097) AAATGTCTTTAAAGATATAAAAATGTCTTTGCGAAAACAGCGAAGACACGTGTCTTTCCCGTTGAATGCACTAAATAACTTGTTTT-TTTTTTTTAATGTTTTTTAAAAACAAATTTGAA
     Ts-1  (2386) AAATGTCTTTAAAGATACAAAAATGTCTTTGCGAAAACAGCGAAGACACGTGTCTTTCCCGTTGAATGCACTAAATAACTTGTTTTCTTTTTTTTAATGTTTTTTAAAAACAAATTTGAA
    Tsu-1  (2427) AAATGTCTTTAAAGATACAAAAATGTCTTTGCGAAAACAGCGAAGACACGTGTCTTTCCTGTTGAATGCACTAAATAACTTGTTTTCTTTTTTTTAATGTTTTTTAAAAACAAATTTGAA


                  3241                                                                                                                3360
    Col-0  (3216) AACACTAACTTTTTTGTGTGATGAAAATGGTCTAAAGAACATCCTCATTAAACAACTTTTAAAAGATGTCTTTAGTATTAATTAGTAATAAAATTTAAAACACTCCTTCTTTTTTGTGTC
     Ts-1  (2506) AACACTAACTTTTTTGTGTGATGAAAATGGTCTAAAGAACATCCTCATTAAACAACTTTTAAAAGATGTCTTTACTATTAATTAGTAATAAAATTTAAAACACTCCTTCTTTTTTGTGTC
    Tsu-1  (2547) AACACTAACTTTTTTGTGTGATGAAAATGGTCTAAAGAACATCCTCATTAAACAACTTTTAAAAGATGTCTTTAGTATTAATTAGTAATAAAATTTAAAACACTCCTTCTTTTGTGTGTC

                  3361                                                                                                                3480
    Col-0  (3336) TAAAATGTCTTTTTAAAGACAAAAACTAAAACTAAAAAACACTCACCATTTTTTATTGATTATTTTTATTAGTTTTATATTTAAAAACACTAATATGGATGTTCTTAGAATATGGTCATC
     Ts-1  (2626) TAAAATGTCTTTTTTAAGACAAAATCTAAAACTAAAAAACACCCACCATTTTTTATTGATTATTTTTATTATTTTTATATTTAAAAACACCAATATGGATGTTCTTAGAGTATGGTCATC
    Tsu-1  (2667) TAAAATGTCTTTTTAAAGACAAAATCTAAAACTAAAAAACACTCACCATTTTTTATTGATTATTTTTATTAGTTTTATATTTAAAAACACCAATATGGATGTTCTTAGAATATGGTCATC

                  3481                                                                                                                3600
    Col-0  (3456) TATATTTCTCAAATAGTTTAATGTGGGTGCTCTTAGAGTATGGTCATTTATATTTTCTCAAATAGTTTTATTTTGTCAACGTTTGGTGTATCAACTATTTTGCAAATAGCAGATACTTTC
     Ts-1  (2746) TATATTTCTCAAATAGTTTAATGTGGGTGCTCTTAGAGTATGGTCATTTATATTTTCTCAAATATTTTTGTTTTGTCAACGTTTGGTGTATCAACTATTTTGCAAATAGCAGATACTTTC
    Tsu-1  (2787) TATATTTCTCAAATAGTTTAATGTGGGTGCTCTTAGACTATGGTCATTTATATTTTCTCAAATAGTTTTATTTTGTCAACGTTTGGTGTATCAACTATTTTGCAAATAGCAGATACTTTC

                  3601                                                                                                                3720
    Col-0  (3576) AGTGAAATTCCTTATCATCGAGGTCATACCTCCTTTGGCATACATTGTAAAGTCTCTAACATAAGCCAACAACTTGCAAGCTCTAATGATTCACAAGTTGATAACACCATTTTGCAAGCT
     Ts-1  (2866) AGTGAAATTCCTTATCATCGAGGTCATACCTCCTTTGGCATACATAGTAAAGTCTCTAACATAAGCCAACAACTTGCAAGCTCTAATGATTCACAAGTTGATAACACCATTTTGCAAGCT
    Tsu-1  (2907) AGTGAAATTCCTTATCATCGAGGTCATACCTCCTTTGGCATACATTGTAAAGTCTCTAACATAAGCCAACAACTTGCAAGCTCTAATGATTCACAAGTTGATAACACCATTTTGCAAGCT

                  3721                                                                                                                3840
    Col-0  (3696) CTAATAATGATTCATTAAACAAATTGGCAATTTTCAAATACCAACACCACCCTTTTTCTTGTTAAAATTACTATTGTTTATTTTATTTTATTTAATGTTAAATTATATTTAAAAGAAAAA
     Ts-1  (2986) CTAATAATGATTCATTAAACAAATTGGCAATTTTCAAATACCAACACCACCCTTTTTCTTGTTAAAATTACTATTGTTTATTTTATTTTATTTAATGTTAAATTATATTTAAAAGAAAAA
    Tsu-1  (3027) CTAATAATGATTCATTAAACAAATTGGCAATTTTCAAATACCAACACCACCCTTTTTCTTGTTAAAATTACTATTGTTTATTTTATTTTATTTAATGTTAAATTATATTTAAAAGAAAAA

                  3841                                                                                                                3960
    Col-0  (3816) TAACCTTGGTACATATGAAATGCTAACTTTTTCAAGAGTTATTTTAAAAAAACAGAATTTCTAATATATCTATTGTCTTGATTCAAACCAAATTTGGATGCCATTTTTGAACTTCAATCC
     Ts-1  (3106) TAACCTTGGTACATATGAAATGCTAACTTTTTCAAGAATTATTTTAAAAAAACAGAATTTCTAATATATCTATTGTCTTGATTCAAACCAAATTTGGATGCCATTTTTGAACTTCAATCC
    Tsu-1  (3147) TAACCTTGGTACATATGAAATGCTAACTTTTTCAAGAATTATTTTAAAAAAACAGAATTTCTAATATATCTATTGTCTTGATTCAAACCAAATTTGGATGCCATTTTTGAACTTCAATCC

                  3961                                                                                                                4080
    Col-0  (3936) TCCACCACCTTGAATTGTGCTCAAACGGTTTCTAATATTCTTGTCTATGATCATCACAAGTATTTCCGTTGGTGATGATTGCTCCCCATGCCTCCTCCTATTCTGTTCATTCCATATTGA
     Ts-1  (3226) TCCACCACCTTGAATTGTGCTCAAACGGTTTCTAATATTCTTGTCTATGATCATCACAAGTATTTCCGTTGGTGATGATTGCTCCC-ATGCCTCCTCCTATTCTGTTCATTCCATATTGA
    Tsu-1  (3267) TCCACCACCTTGAATTGTGCTCAAACGGTTTCTAATATTCTTGTCTATGATCATCACAAGTATTTCCGTTGGTGATGATTGCTCCCCATGCCTCCTCCTATTCTGTTCATTCCATATTGA

                  4081                                                                                                                4200
    Col-0  (4056) GTGTATAGAGTTCTGTAGTGCATACCGAAGTATGTAGCTCTTCGTTTTGTCCACAAGGTTTTCAGATAAAATCTTCATAACCTCTGTCCAACTCACCGAGAATCTGTCCGAGAAAATTCC
     Ts-1  (3345) GTGTATAGAGTTCTGTAGTGCATACCGAAGTATGTAGCTCTTCGTTTTGTCCACAAGGTTTTCAGATAAAATCTTCATAACCTCTATCCAACTCACCGAGAATCTGTCCGAGAAAATTCC
    Tsu-1  (3387) GTGTATAGAGTTCTGTAGTGCATACCGAAGTATGTAGCTCTTCGTTTTGTCCACAAGGTTTTCAGATAAAATCTTCATAACCTCTGTCCAACTCACCGAGAATCTGTCCGAGAAAATTCC

                  4201                                                                                                                4320
    Col-0  (4176) CCTTGTTAGGTCTTCCCATATCTTTTTGAGTATAGGCAGTGGAAAAATAAGTGTTCCCTCGTCTCTACTCG--------------TTCATTGCAAAAAATGCAGCTCGAATCTACATTAC
     Ts-1  (3465) CCTTGTTAGGTCTTCCCATATCTTTTTGAGTAGAGGCAGTGGAAAAATAAGTGTTCCCTCGTCTCTACTTGTTCATTGCAAATTGTTCATTGCAAAAAATGCAGCTCGAATCTACATTAC
    Tsu-1  (3507) CCTTGTTAGGTCTTCCCATATCTTTTTGAGTAGAGGCAGTGGAAAAATAAGTGTTCCCTCGTCTCTACTTGCTCATTGCAAATTGTTCATTGCAAAAATTGCAGCTCGAATCTACATTAC

                  4321                                                                                                                4440
    Col-0  (4282) AATTCCACTTCCTCATTCTCTCCCCTGTTGCGACTCTGTTTTTTACAACCGTATTGTTATACTTTGGAGTAGCGTATGGGAACCATATCTCATTTTGTCCCTCCATCGGCGGCTTTGCTA
     Ts-1  (3585) AATTCCACTTCCTCATTCTCTCCCCTGTTGCGACTCTGTTTTTTACAACCGTATTGTTATACTTTGGAGTAGCGTATGGGAACCATATCTCATTTTGTCCCTCCATCGGCGGCTTTGCTA
    Tsu-1  (3627) AACTCCACTTCCTCATTCTCTCCCCTGTTGCGACTCTGTTTTTTACAACCGTATTGTTATACTTTGGAGTAGCGTATGGGAACCATATCTCATTTTGTCCCTCCATCGGCGGCTTTGCTA

                  4441                                                                                                                4560
    Col-0  (4402) ACCGCAGCTGTGACCAAGAACATTTATTTTTCCCTTTTAACAGAACTATATCGTGATCGTTCAGTTCTCCATTCAGCTTCTGTTTTCTTATTTCTTCTTCAACACGGTTTAAGATACCCT
     Ts-1  (3705) ACCGCAGCTGTGACCAAGAACATTTATTTTTCCCTTTTAACAGAACTATATCGTGATCGTTCAGTTCTCCATTCAGCTTCTGTTTTCTTATTTCTTCTTCAACACGGTTTAAGATACCCT
    Tsu-1  (3747) ACCGCAGCTGTGACCAAGAACATTTATTTTTCCCTTTTAACAGAACTATATCGTGATCGTTCAGTTCTCCATTCAGCTTCTGTTTTCTTATTTCTTCTTCAACACGGTTTAAGATACCCT

                  4561                                                                                                                4680
    Col-0  (4522) TTCGATGGTTTCTCCTTCGTGTCATGGACATCACCTCTTTCATTGTGCTTGAACTAGGAATCCCTAGATCAATGCATCCTCTCTAAGACATCCCATGTTACTCCATGTGTCAATACCAAA
     Ts-1  (3825) TTCGATGGTTTCTCCTTCGTGTCAGGGACATCACCTCTTTCATTGTGCTTGAACTAGGAATCCCTAGATCAATGCATCCTCTCTAAGACATCCCATGTTACTCCATGTGTCAATACCAAA
    Tsu-1  (3867) TTCGATGGTTTCTCCTTCGTGTCATGGACATCACCTCTTTCATTGTGCTTGAACTAGGAATCCCTAGATCAATGCATCCTCTCTAAGACATCCCATGTTACTCCATGTGTCAATACCAAA

                  4681                                                                                                                4800
    Col-0  (4642) AAGACGTAGTTTCGCCGCTTTTAACCACTACCATGACTGTTGATCTCTTTAAATAATTAAAAAGTGGTGTTCCCCATGAAGACCGTTCACTTCCATCAATTTATGGTCTTCTT-GGACCA
     Ts-1  (3945) AAGACGTAGTTTCGCCGCTTTTAACCACTACCATGACTGTTGATCTCTTTAAATAATTAAAAAGTGGTGTTCCCCATGAGGACCGTTCACTTCCATCAATTTATGGTCTTCTT-GGACCA
    Tsu-1  (3987) AAGACGTAGTTTCGCCGCTTTTAACCACTACCATGACTGTTGATCTCTTTAAATAATTAAAAAGTGGTGTTCCCCATGAGGACCGTTCACTTCCATCAATTTATGGTCTTCTTTGGACCA

                  4801                                                                                                                4920
    Col-0  (4761) ATTTCGTTTATCCAAACTATTACCCCTACAACATCGTTTATAAATTGTCTTT-ACATTTTGATCTAGAATTTGATCTATTTTGCACGAATCCTCTTCCACCTTTTCAGGCATGTACGAGC
     Ts-1  (4064) ATTTCGTTTATCCAAACTATTACCCCTACAACATCGTTTATAAATTGTCTTT-ACATTTTGATCTAGAATTTGATCTATTTTGCACGAATCCTCTTCCACCTTTTCAGGCATGTACGAGC
    Tsu-1  (4107) ATTTCGTTTATCCAAACTATTACCCCTACAACATCGTTTATAAATTGTCTTTTACATTTTGATCTAGAATTTGATCTATTTTGCACGAATCCTCTTCCACCTTTTCAGGCATGTACGAGC

                  4921                                                                                                                5040
    Col-0  (4880) AATATAACATGAGTTTGATCAAATTTTGCATGGTCTCTACGAAGTTGAGTCATGCAATTTTTACCTATAATTTTTGTTCTCGGCATTTTTATAGCTCTCTACTTAGGATATAAGTAACAT
     Ts-1  (4183) AATATAACATGAGTTTGATCAAATTTTGCATGGTCTCTACGAAGTTGAGTCATGCAATTTTTACCTATAATTTTTGTTCTCGGCATTTTTATAGCTCTCTACTTAGGATATAAGTAACAT
    Tsu-1  (4227) AATATAACATGAGTTTGATCAAATTTTGCATGGTCTCTACGAAGTTGAGTCATGCAATTTTTACCTATAATTTTTGTTCTCGGCATTTTTATAGCTCTCTACTTAGGATATAAGTAACAT

                  5041                                                                                                                5160
    Col-0  (5000) GGAGTTGGATGAATCCTTTTCTTTATATTGAAATCGAATCTTCAATGCTAAAAATAAAATCCACTCAATCAGTGAGAGAATTATTATGGAACAAGCTATA----AAGACGAAAGAATCAC
     Ts-1  (4303) GGAGTTGGATGAATCCTTTTCTTTATATTGAAATCGAATCTTCAATGCTAAAAATAAAATCCACTCAATCAGTGAGAGAATTATTATGGAACAAACTATATATAAAGACGAAAGAATCAC
    Tsu-1  (4347) GGAGTTGGATGAATCCTTTTCTTTATATTGAAATCGAATCTTCAATGCTAAAAATAAAATCCACTCAATCAGTGAGAGAATTATTATGGAACAAACTATATATAAAGACGAAAGAATCAC

                  5161                                                                                                                5280
    Col-0  (5116) TCAATCATACAATAAAGAAGGATGTTTTTTTCATTTAAACTAAGAAAGAGAACTTTTCCACGTGAATTAAAATAGACATCTCAATAATAAAGACGATTTTTTTTCTTCTCTTTTTCCTCT
     Ts-1  (4423) TCAATCATACAATAAAGAAGGATGTTTTTTTCATTTAAATTAAGAAAGAGAACTTTTCCACGTGAATTAAAATAGACATCTCAATAATAAAGACGATTTTTTTT-CTCTCTTTTTCCTCT
```

Tsu-1 (4467) TCAATCATACAATAAAGAAGGATGTTTTTTTCATTTAAATTAAGAAAGAGAACTTTTCCACGTGAATTAAAATAGACATCTCAATAATAAAGACGATTTTTTTTTCTCTCTTTTTCCTCT

```
                  5281                                                                                                                5400
    Col-0  (5236) CACTTTGTATTGTATTGTATTTCTATATATTTCTTCCTCTTACAAATACCCTTTTAAATTGGAAAGAAAAAA---CAGGAAT-CGCTATCATCAGTAATAGTC-ATCATTAATCA-ATTT
     Ts-1  (4542) CACTTTGTATTGTA-----TTTCTATATCTTTCTTCCTCTTACAAATACCCTTTTAAATTGGAAAGAAAAAATCTCTGAAAAACGATATAAACTGGACAACGCTATCATTAGTAATAGTA
    Tsu-1  (4587) CACTTTGTATTGTA-----TTTCTATATCTTTCTTCCTCTTACAAATACCCTTTTAAATTGGAAAGAAAAAATCTCTGAAAAACGATATAAACTGGACAACGCTATCATTAGTAATAGTA

                  5401                                                                                                      5510
    Col-0  (5350) AT-ATGTAATATGTGGCTGACAATTTCCATGTACGTGTAATATGTAATATATAAACACAACTTATGGCCAGTATAATATTAATGCTTAAACCGACTCGAGAACTAAAATG
     Ts-1  (4657) ATCATCTTTTATGTGGCTGACAATTTCCATGTACGTGTAATATGTAATATATAAACACAA-TCATGGCCAGTATAATATTAATGCTTAGACCGACTCGAGAACTAAAATG
    Tsu-1  (4702) ATCATTTTTTATGTGGCTGACAATTTCCATGTACGTGTAATATGTAATATATAAACACAA-TCATGGCCAGTATAATATTAATGCTTAGACCGACTCGAGAACTAAAATG
```

(*) Major polymorphisms between Col-0 and Ts-1 and Tsu-1 sequences have been highlighted.

1. The first major polymorphism affects two repeated units that are present in Col-0 (Letters in blue).

2. In red are represented in Ts-1 and Tsu-1 duplicated sequences that are only present once in Col-0.

3. The putative CAAT and TATA boxes predicted by Uozumi *et al.* (2000) as well as the ATG translational start codon have been underlined. The first CAAT box is not present in the Ts-1 and Tsu-1 promoter sequences as a consequence of the polymorphism.

4. Confirmed SNPs as well as small Indels between Col-0, Ts-1 and Tsu-1 sequences appear in bold letters.
