# Supplementary material for: Natural Variants of AtHKT1 Enhance Na+ Accumulation in Two Wild Populations of Arabidopsis
Source: PLoS Genet. 2006 Dec 1;2(12):e210. doi: 10.1371/journal.pgen.0020210 (PMC1665649; doi:10.1371/journal.pgen.0020210)
Supplement: Supplementary file 2 [file pgen.0020210.st001.doc]

Table S1: List of pairs of primers used to sequence the At*HKT1* gene and PCR product sizes expected for Col-0 and obtained for Ts-1 and Tsu-1.

| Primer pairs | Primer sequences | PCR product size (bp) | | |
| --- | --- | --- | --- | --- |
|  |  | Col-0 | Ts-1 | Tsu-1 |
| HKT1-F1  HKT1-R1 | 5’-AGAAATCGCTCAACAAACTAC-3’  5’-AAAATGTTATCTCCACCTAGG-3’ | 630 | 612 | 653 |
| HKT1-F2  HKT1-R2 | 5’-TTA TCT CTT AGA AGA TCT CCG-3’  5’-AGT ATC GAG GAA AAG TAA CAC-3’ | 689 | No amplification | No amplification |
| HKT1-F3  HKT1-R3 | 5’-AAACACTAAAAGAGAGGTCAC-3’  5’-TGGTTGAAAATATGTACGAACAC-3’ | 537 | 537 | 534 |
| HKT1-F1  HKT1-R3 | 5’-AGAAATCGCTCAACAAACTAC-3’  5’-TGGTTGAAAATATGTACGAACAC-3’ | 1,655 | 930 | 971 |
| HKT1-F4  HKT1-R4 | 5’-ATATCACTTCCTAGATTGTGTC-3’  5’-ACTCACATACAAACTGACAATG-3’ | 707 | 708 | 708 |
| HKT1-F5  HKT1-R5 | 5’-AAAACATCCACATTGGTGTAC-3’  5’-TGATTCTTTCTTGGAATCTCC-3’ | 605 | 618 | 618 |
| HKT1-F6  HKT1-R6 | 5’-ACGTGTAATTCACAAAAGTAGC-3’  5’-TTACATGGTTAATGAATACCTC-3’ | 494 | 494 | 494 |
| HKT1-F7  HKT1-R7 | 5’-AAAAAGAGTGTACTCTGACAC-3’  5’-TGGCTTATGTTAGAGACTTTAC-3’ | 660 | 661 | 661 |
| HKT1-F8  HKT1-R8 | 5’-TTTGTCAACGTTTGGTGTATC-3’  5’-AATTTTCTCGGACAGATTCTC-3’ | 647 | 646 | 647 |
| HKT1-F9  HKT1-R9 | 5’-TAGAGTTCTGTAGTGCATACC-3’  5’-TAAATTGATGGAAGTGAACGG-3’ | 684 | 698 | 698 |
| HKT1-F10  HKT1-R10-2 | 5’-TAAGACATCCCATGTTACTCC-3’  5’-TACACGTACATGGAAATTGTC-3’ | 783 | 788 | 791 |
| HKT1-F11  HKT1-R11 | 5’-TCTTCCTCTTACAAATACCC-3’  5’-AAGAACCACCGAGTACAAGC-3’ | 675 | 681 | 681 |
| HKT1-F12  HKT1-R12 | 5’-TGACGTTGAGACTGTTACTG-3’  5’-AGAGTACCAAGATAGCTGGG-3’ | 659 | 659 | 659 |
| HKT1-F13  HKT1-R13 | 5’-ATACAGCTTCTTTTCTTCTGC-3’  5’-TTACTTCCTCAATCCATGGGAG-3’ | 790 | 791 | 789 |
| HKT1-F14  HKT1-R14 | 5’-TTAGTGCTATTCTTTTGGCTG-3’  5’-TACTCTTAAGTGATGGAGAG-3’ | 531 | 530 | 530 |
| HKT1-F15  HKT1-R15 | 5’-AAACATTAACGCCGGAAAAC-3’  5’-CTTTCGGTGATTGAAATGAG-3’ | 559 | 558 | 559 |
| HKT1-F16  HKT1-R16-3 | 5’-AGGAGGAGATGATGATTCCG-3’  5’-TTAATAAATTCTTCCGGTCCC-3’ | 881 | 882 | 881 |
| HKT1-F18  HKT1-R18 | 5’-TTTTATACTACTCCCAACTCG-3’  5’-AACGAGGATTAACGATGATGC-3’ | 526 | No amplification | No amplification |
| HKT1-F19-1  HKT1-R18 | 5’-TAATGGCAGTGCATATGGAAAC-3’  5’-AACGAGGATTAACGATGATGC-3’ | 337 | 337 | 337 |
| HKT1-F19  HKT1-R19 | 5’-TATGGTAGGTTTAAGCAGTTC-3’  5’-AAGAAGACATGTGTGTCAAAC-3’ | 455 | 315 | 315 |
